# Triangulation in aetiological epidemiology

**DOI:** 10.1093/ije/dyw314

**Published:** 2017-01-20

**Authors:** Debbie A Lawlor, Kate Tilling, George Davey Smith

**Affiliations:** 1 MRC Integrative Epidemiology Unit at the University of Bristol, Bristol, UK; 2 School of Social and Community Medicine, University of Bristol, Bristol, UK

**Keywords:** Aetiological epidemiology, causality, instrumental variables, Mendelian randomization, natural experiments, negative control studies, RCTs, triangulation, within-sibships studies

## Abstract

Triangulation is the practice of obtaining more reliable answers to research questions through integrating results from several different approaches, where each approach has different key sources of potential bias that are unrelated to each other. With respect to causal questions in aetiological epidemiology, if the results of different approaches all point to the same conclusion, this strengthens confidence in the finding. This is particularly the case when the key sources of bias of some of the approaches would predict that findings would point in opposite directions if they were due to such biases. Where there are inconsistencies, understanding the key sources of bias of each approach can help to identify what further research is required to address the causal question. The aim of this paper is to illustrate how triangulation might be used to improve causal inference in aetiological epidemiology. We propose a minimum set of criteria for use in triangulation in aetiological epidemiology, summarize the key sources of bias of several approaches and describe how these might be integrated within a triangulation framework. We emphasize the importance of being explicit about the expected direction of bias within each approach, whenever this is possible, and seeking to identify approaches that would be expected to bias the true causal effect in different directions. We also note the importance, when comparing results, of taking account of differences in the duration and timing of exposures. We provide three examples to illustrate these points.

## Introduction

Aetiological epidemiology–understanding what causes differing levels of disease in populations–is central to the science of epidemiology. However, there is considerable debate about the circumstances under which causality can be tested or assumed.^1–6^ Well-conducted randomized controlled trials (RCTs) provide the best causal evidence for treatment effectiveness, but they are not always feasible or ethical. For example, the causal effects of circumstances that influence social justice,^6^ or behaviours such as breastfeeding, alcohol consumption and smoking, particularly when the interest is in long-term effects of these exposures,^7^ are difficult (or impossible) to test with RCTs. Furthermore, whether we are interested in the causal effect of an exposure that may be modified by a complex population health intervention, or one that would be the target for a drug intervention, the cost of developing and trialling those interventions highlights the importance of obtaining the best causal evidence about exposures before proceeding to intervention development and RCTs.

The aim of this paper is to illustrate how triangulation–the integration of evidence from several different epidemiological approaches that have differing and unrelated key sources of bias–might be used to improve causal inference in aetiological epidemiology. The word ‘approaches’ rather than methods or study design is intentionally used because comparisons may be made between different study designs (e.g. between RCTs and cohort or cross-sectional studies), and/or between different analytical approaches within the same study design (e.g. between negative control studies, cross-context comparisons or Mendelian randomization (MR), all undertaken in cohort study designs).

## Definition of triangulation

Triangulation has been used in quantitative surveying from at least the 1600s to describe a method that calculates a distance that is difficult (or impossible) to measure, from two or more easier-to-measure distances.^8^^,^^9^ The distance of interest is calculated by using the known mathematical properties of triangles. The more recent use of the term in qualitative and quantitative research–to describe obtaining more reliable or accurate answers to research questions by comparing results from two or more different study approaches–has been criticized for being unrelated to the original use of the term, and for not having clear definitions or criteria for its use.^10–13^ We believe that when the term ‘triangulation’ is used in research, its meaning is similar to its original use in surveying. In both there is a ‘measure’ that cannot be (easily) obtained (in aetiological epidemiology this would be an unbiased causal effect estimate), and you estimate that measure from different locations (in aetiological epidemiology, from different approaches with different sources of bias). Nonetheless, we agree that a clear definition and criteria for its use in research are important.

Triangulation is used in many research fields, including sociology, education, theoretical physics and mathematics,^11^^,^^13^^,^^14^ and much of what we write might have relevance for other disciplines. However, our focus in this paper is on aetiological epidemiology and the integration of different epidemiological approaches in a triangulation framework. We propose the following definition of triangulation in aetiological epidemiology:

*The practice of strengthening causal inferences by integrating results from several different approaches, where each approach has different (and assumed to be largely unrelated) key sources of potential bias*.

Other commonly used methods of integrating epidemiological evidence, such as independent replication or validation, meta-analysis and systematic reviews, seek to compare (and, in some cases, quantitatively combine) results from the same study design/approach under the assumption that they are all unbiased and estimating the same underlying causal effect. Triangulation aims to integrate data from different methodological approaches with different biases and to exploit these differences to draw qualitative conclusions. The idea behind triangulation is that when we compare different approaches with assumed unrelated sources of bias, particularly if the expected direction of bias for some of the approaches is different, we would not expect to obtain the same estimates of the causal effect (unless all were unbiased).^7^^,^^15^ Thus, triangulation has some features in common with Austin Bradford Hill’s concept of ‘consistency’ which he defines in his considerations on causality as ‘[results that have] been repeatedly observed by different persons, in different places, circumstances and times’.^16^

## Criteria for triangulation in aetiological epidemiology

We propose that the following minimum set of criteria should be fulfilled for triangulation to be valid in aetiological epidemiology.

- Results from at least two, but ideally more, different approaches, with differing and unrelated key sources of potential biases, are compared.
- The different approaches address the same underlying causal question.
- Related to the above criterion, for each approach the duration and timing of exposure that it assesses is taken into account when comparing results.
- For each approach the key sources of bias are explicitly acknowledged when comparing results.
- For each approach the expected direction of all key sources of potential bias are made explicit where this is feasible, and ideally within the set of approaches being compared there are approaches with potential biases that are in opposite directions.

In the Table 1 we summarize the key sources of bias for several ‘conventional’ and also some additional aetiological approaches that might be used in triangulation. The additional approaches are not all ‘novel’, in that several have been in use for many years, but their use is not yet widespread. These additional methods are not inherently different from the ‘conventional’ approaches and several could be applied to the same dataset. They represent refinements of conventional approaches, intended to specifically explore and assess different possible sources of bias, by varying the population(s), exposures and/or outcomes under study. For each approach we provide a brief summary of the method, assumptions and key sources of bias (Table 1) together with illustrative examples of their use to address aetiological questions (Table S1, available as Supplementary data at *IJE* online). Providing detailed descriptions of each of these approaches is beyond the scope of this paper; readers are referred to other publications for more information on them.^17–42^ We have included three different uses of instrumental variable (IV) analyses in Table 1 (use to test intermediates in an RCT, genetic (MR) and non-genetic IVs in observational data) because these will potentially have different key sources of bias. It would not be possible to include every possible approach that might be used in triangulation in Table 1. We have selected approaches that we believe are sufficiently different that they are likely to have different key sources bias.

**Table 1.** Key sources of bias in different aetiological epidemiology approaches

**Approach**	**Description**	**Assumptions**	**General key sources of potential bias**^a^
**Conventional approaches**			
Randomized controlled trials^17^^,^^18^	Prospective intervention study in which people are randomly allocated to comparison groups that are given different interventions	Intervention groups are similar with the exception of the intervention	Lack of concealment of the random allocation. Failure to maintain the original randomized status of participants when comparing outcomes and lack of blinding to which group participants have been randomised. Differential loss to follow-up, for example due to adverse effects of the intervention or a perception that there is no benefit
Multivariable regression in observational data^19^^,^^20^	The application of multivariable regression to observational data	No residual confounding (all confounders are accurately measured and controlled for). Participants are not selected to participate or to be included in analyses in a way that produces a spurious associations. Any misclassification of exposure is not related to the outcome, and vice versa, and misclassification of covariables are not systematically related to outcome or exposure	Unmeasured or poorly measured confounders (residual confounding). Reverse causality. Misclassification of exposure is related to the outcome (or vice versa). Differential missing data between exposure levels, for example due loss to follow-up in prospective cohort studies or reporting bias in case-control studies
**Refinements using general populations**			
Cross-context comparisons^21^	Compares results between two or more populations in different contexts that result in confounding structures being different	Different results between populations are due to different confounding structures and not due to true differences in causal effects between populations. Similar results between populations cannot be explained by confounding, given the differences between the populations/contexts in their confounding structures. There are no other (than confounding) sources of bias that could explain similar or different results between the two populations	Confounders are, in fact, the same in the populations being compared. For observed confounders, differences between the two populations should be established. There are different sources of bias (over and above different confounding structures), for example differential misclassification of exposure or outcome that investigators are unaware of. Measurement of the exposure and outcome and the quality of these measurements should be the same or very similar in the populations being compared
Different control groups^22–24^	Use of two or more different control groups in a case-control study, where the bias for the different control groups is expected to be in different directions	The different sources of bias for the different control groups are different and would produce different results	If biases are in fact the same in the different control groups being compared, the inference made when comparing them will be misleading. If there are different sources of bias between the different control groups, but these nonetheless distort the finding in the same direction, this will also be misleading. Inference may be incorrect, if there are a priori incorrect assumptions about one of the control groups being least biased for the specific research question. This may be less statistically efficient than having just one control group, as with fixed resources it would imply using a smaller number of controls for each of the two groups (and possibly a smaller number of cases, as resources would be required to recruit two different sources of controls)
Natural experiments^25^	Populations are compared in the belief that biases, such as confounding structures are similar between them. One (or more) of the populations has had a ‘natural’ exposure or are ‘quasi-randomly’ exposed. Natural exposure, e.g. flood or famine; quasi- randomization, e.g. those resulting from different timing of introduction to policies, such as smoking bans in public places	The populations being compared are similar with the exception of the naturally or quasi-randomized exposure	Populations differ on characteristics that confound the association. Misclassification of the outcome is related to the naturally occurring exposure. Ideally, identical methods for measuring the outcome should be used in each population. If associations are measured at the aggregated population level but interpreted as if they apply to individuals within the population, there may be bias due to the ecological fallacy
**Refinements using specific populations**			
Within sibling comparisons^26–30^	Assesses associations within sibships: comparing outcomes between sibs who are discordant for the exposure. Controls for observed and unobserved shared (familial) confounding	There is little or no individual-level confounding. Any misclassification of exposure or outcome is similar in the siblings	Individual level confounding could occur when siblings are raised in different environments. This approach works best when there is strong family-level confounding, with modest main effects, or where correlation within sibships is much stronger for the confounders than it is for the exposure of interest. This may be the case when examining the effect of intrauterine or early infancy exposures on outcomes assessed several years later.^27–29^ In within-sibship analyses, individual confounding will produce greater bias than in equivalent studies examining associations between unrelated people, because only siblings who are discordant for the exposure are included in analyses, and family-shared causes of the exposure cannot cause this discordancy.^30^ Similarly, bias due to misclassification of the exposure (or outcome) will be greater than that seen in studies of unrelated individuals^30^
**Refinements of exposure**			
Instrumental variable (IV) analyses^31^	IVs are variables that are robustly associated with an exposure but not with confounders of the exposure and outcome (Figure 1).	IV is associated with exposure. IV is not associated with confounders of exposure-outcome association. IV is not related to the outcome other than via its association with the exposure (the exclusion restriction criteria)	IV is not truly associated with exposure in the population being studied. There should be robust evidence (e.g. replicated in several different studies) that the IV is related to the exposure, and ideally its association in the study population should be established. If the statistical magnitude of association of the IV with exposure in a study is small, there may be weak instrument bias which would bias towards the results of the confounded exposure-outcome association in one-sample IV analyses and towards the null with two-sample IVs^35^^,^^38^

IVs to test intermediates in RCTs^32^	IV is randomization to an intervention that affects an intermediate of the randomized intervention; this intermediate is the exposure of interest (e.g. shown in Figure 1)	As above	Violation of the exclusion restriction criteria is likely to be the main source of bias. Comparing results from multiple IVs that work in different ways to affect the intermediate (e.g. comparing results from RCTs to different antihypertensives to determine the causal effect of BP on CHD^32^). When both this approach and MR are triangulated for the same causal question, the source of violation of this assumption might be different (in which case triangulation will be valid) or might the same (triangulation would not be valid) (see Figure 1 and section on ‘What we mean by unrelated sources of bias’). Weak instrument bias might is also a potential key source of bias in this use of IVs. In well-conducted RCTs, it is unlikely that the IV will be related to confounders
Genetic IVs in observational data (MR)^33–38^	IV is one or more genetic variant(s) that have been shown to robustly relate to exposure	As above	Violation of the exclusion restriction criteria, as a result of genuine (also known as horizontal) pleiotropy (Figure 1) is likely to be the main source of bias.^35–38^ Using multiple genetic IVs that likely have different (unrelated) paths to the exposure, and employing recently developed sensitivity analyses to these, can test and control (to some extent) for this violation^36^^,^^37^ Population stratification produces confounding. This may be avoided by using ethnically homogeneous populations and/or controlling for principal components that reflect different population subgroups.^33–35^ With increasing availability of results from large-scale genome-wide association studies and application of two-sample MR to these. weak instrument bias is less likely and when it occurs would bias towards the null^35^^,^^38^
Non-genetic IVs in observational data^25^	IV is non-genetic, examples include use of exposures in other family members as IVs for the index participants’ exposure, or a ‘natural’ occurring phenomenon (such as famine or flood); this approach is commonly used in natural experiments^25^	As above	Association of the IV with confounders of the exposure-outcome association are more likely with this approach than IVs for intermediates in an RCT or MR. Violation of the exclusion restriction criteria is possible; given the wide range of non-genetic IVs that could potentially be used, the extent to which this may be a major source of bias is hard to state in a general way. Weak instrument bias is possible.
Exposure negative control studies^39–42^	Aims to reproduce the same conditions as the ‘real’ study, but uses a different (negative control) exposure that is not plausibly causally related to outcome	The key sources of bias, including specific confounders, misclassification bias and other biases, are the same for the real and negative control exposures. The negative control exposure does not have a causal effect on the real outcome. To sensibly compare the real and negative control exposure, they should ideally be similarly scaled. This should be possible when negative control exposures are used to test critical or sensitive periods (see section on duration and timing of exposure being assessed with different exposures)	There are differences in the sources of bias between the real and negative control exposure. Attempts to explore this (e.g. exploring the association of observed confounders with the negative control exposure) should be made. There is a real (but unknown) causal effect of the negative control exposure on the outcome
**Refinements of outcome**			
Outcome negative control studies^39–42^	As above, except here a different outcome is selected for the negative control study	As above, except here a different outcome is selected for the negative control study	As above, if either assumption is violated there could be biased inference from the comparison of the real with the negative control study

a We have tried to list most of the key sources of bias for different approaches, but the extent to which these are a key bias in any given triangulation example will depend upon the question being asked and the approaches and data being used to answer this. For example, in general, violation of the exclusion restriction criteria will be a key source of bias in MR studies and use of IVs to test intermediates in RCT; but as we discuss in the section on ‘What we mean by key sources of bias’, sometimes the source will be the same for these two approaches and sometimes it will not. Furthermore, in the second illustrative example, whereas we recognize that violation of the exclusion restriction criteria might bias the IV testing of glucose effects in the RCT, the assumptions we had to make about change in glucose in the control arm are (in that specific example) likely to be a bigger source of bias. The direction of any bias will depend on the question being asked and the approaches and data being used.

There are a number of different statistical methods that, like multivariable regression, can be used to control for observed confounders, such as stratifying, propensity scores, inverse probability weighting, g-computation and parametric g-formula. Other methods, like instrumental variable analyses and matching methods, aim to control for observed and unobserved confounders; these include regression discontinuity and difference in differences. These different analytical methods could all be applied to the same dataset. However, we think it important to note that this would not be triangulation as defined here. These statistical methods have different underlying statistical assumptions, but we do not feel that these result in different key sources of bias as do the approaches we list in Table 1. For example, key sources of biases from these different methods applied to the same data set would be biased by the same measurement error and residual confounding due to unobserved and/or poorly measured confounders. Finding similar results from the application of each of them to a dataset and concluding that this similar answer is likely to be the correct causal answer would be clearly wrong; the similar answer is likely to be the (residually) confounded answer. There is value in using several of these statistical methods in aetiological epidemiology to test the sensitivity of results to statistical model assumptions, as done in a recent study of the effect of reductions in housing benefit on mental health in the UK,^43^ but this is not triangulation and we therefore do not consider these different methods further in this paper.

## What we mean by unrelated sources of bias

By ‘sources’ of bias we mean the process through which specific biases might occur. For example, confounding may bias causal estimates from multivariable regression analyses in prospective cohort, RCT and MR studies, but the key source of confounding will differ for each of these approaches. In observational studies, the key source of confounding will be unmeasured or poorly measured confounders.^19^^,^^20^ In RCTs, failure to compare outcomes by the original randomized groups or subversion of the randomized process by researchers or participants is a likely key source of confounding. MR studies are unlikely to be confounded by the many socioeconomic and lifestyle characteristics that plague conventional observational studies,^44^ but may be confounded by population stratification.^33^^,^^34^

Thus, in each of the three approaches (multivariable regression in a prospective cohort, RCT and MR), confounding can compromise causal effect estimation, but its source is markedly different and we would assume in a triangulation study using these approaches that bias from unmeasured confounders, failure to examine effects in randomized groups or subversion of the randomization, and population stratification in an MR study would not be related to each other. Thus, if these three approaches were used to test the same underlying causal question, it is unlikely that they would each produce the same (wrong) answer as a result of confounding.^45^

**Figure 1.**
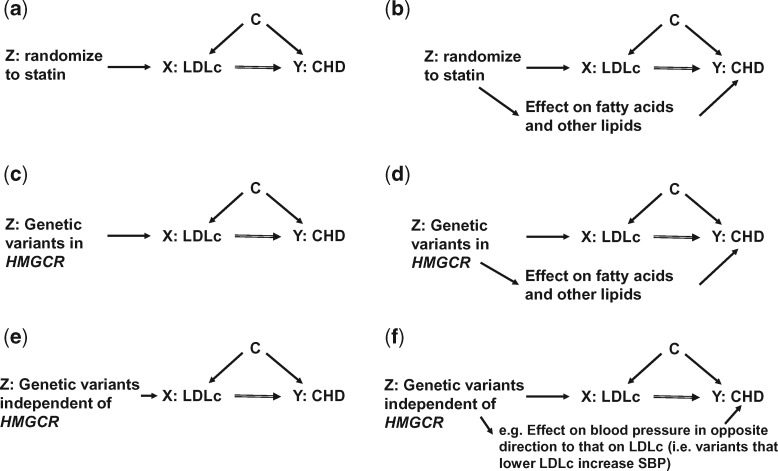
Illustrative example of instrumental variable analyses in RCTs and Mendelian randomization studies to answer aetiological questions of the effect of a risk factor (LDLc) on an outcome (CHD). The figure shows directed acyclic graphs (DAGs) of instrumental variable (IV) analyses to test the causal effect of low-density lipoprotein cholesterol (LDLc) on coronary heart disease (CHD). In a and b, the IV is randomization to receiving a statin or not (i.e. this is an example of IV analyses to test an intermediate in an RCT); statins are 3-hydroxy-3-methylglutaryl-coenzyme (HMG-CoA) reductase inhibitors. In (c) and (d), the IV is genetic variants in the *HMGCR* gene (i.e. this is an MR study); these variants mimic HMG-CoA reductase inhibition. In (e) and (f) the IV is genetic variants (MR) that are independent of those in the *HMGCR* genes. The three key assumptions of IV analyses are illustrated in (a), (c) and (e), that the: (i) IV ‘Z’ (randomization to statins in a and genetic variants related to LDLc in (c) and (e) is (or is plausibly) robustly related to the risk factor (LDLc in all figures); (ii) IV is not related to confounders (shown by letter C in all figures) for the risk factor-outcome association (shown by the lack of an arrow from C to Z in all figures); (iii) IV only affects the outcome ‘Y’ (CHD) through its effect on the risk factor ‘X’ (LDLc). This last assumption is known as the exclusion restriction criteria. In the RCT of statins example, we know that assumption (i) is true, and if the RCT is well conducted then assumption (ii) will be true. If, however, statins are directly (independently of LDLc) related to other factors which then affect CHD, assumption (iii) will be violated and the estimated causal effect a biased estimate of the true effect of LDLc. There is some evidence that statins do relate to a wide range of other lipids and fatty acids in addition to LDLc,^46^ though whether these are caused by the statins independent of LDLc and affect CHD is currently unknown. If they do (as shown as an illustrative example in (b) then the estimate of the LDLc effect on CHD is likely to be biased (what is assumed to be the effect of LDLc on CHD will be the combined effect of LDLc and other lipids/fatty acids on CHD). In the MR example of variants in the *HMGCR* gene, we know that assumption (i) is correct and there is evidence that assumption (ii) is also this is likely to be true.^44^ As with the RCT example, in MR we are often most worried about violation of assumption (iii), due to genuine (horizontal) pleiotropy in MR^35–38^–i.e. that variants in *HMGCR* influence other factors independently of LDLc which in turn (independently of LDLc) affect CHD (d). As these variants are mimicking the action of statins, then any pleiotropy is likely to be similar to that seen for statins^46^ (d). By contrast, (e) and (f) show the use of genetic variants that are unrelated to *HMGCR*. Although there may still be violation of the exclusion restriction criteria (due to genuine pleiotropy) with these variants, it is unlikely to be related to violation of the exclusion restriction criteria in an RCT of statins because the variants have been selected on the basis that their actions are on a different path from those of statins.

Similarly, violation of the ‘exclusion restriction criteria’^33–37^ could bias causal estimates in IV analyses when used to test effects of intermediates in RCTs or when used in observational data (including MR), but if the source of violation of this assumption is different, and unrelated, in each of these approaches it would be appropriate to triangulate them (Figure 1). The exclusion restriction criteria states that the IV, such as randomization to a treatment or a set of genetic variants, does not influence the outcome other than through the risk factor of interest.^33–37^ In some circumstances the key source of violation of the exclusion restriction criteria might be the same, even when the study designs are different. For example, in Figures 1a–d, we show how using randomization to a statin in an RCT to determine the causal effect of low-density lipoprotein cholesterol (LDLc) on coronary heart disease (CHD) might have the same source of violation of this criterion as an MR study that uses (a) genetic variant(s) which influence(s) the same biological path as that which statins act on.^46^ In this case we would want to avoid comparing these two studies in triangulation, as the key source of bias is essentially the same. When an IV to test an intermediate in an RCT and genetic variants in MR are unrelated to each other (as suggested in Figure 1e and f, and in our second illustrative example below), including both approaches (ideally together with other approaches), would be appropriate.

Because MR is relatively novel there has been considerable focus on its limitations and methods for testing and dealing with these, in particular for dealing with violation of the exclusion restriction criteria due to genuine pleiotropy.^36^^,^^37^ Two of these methods in particular (MR-Egger^36^ and the weighted median estimator^37^) are likely to become increasingly used in MR sensitivity analyses, as two-sample MR using summary genome-wide association studies (GWAS) data becomes more common.^38^ In comparison with the conventional MR approach (e.g. the Wald estimator), these methods ‘relax’ some of the underlying IV assumptions in different ways (summarized in Supplementary text available as Supplementary data at *IJE* online) and, as we do in the first example below, when possible we recommend applying both of these methods as sensitivity analyses to MR studies. Although these approaches have been developed for use in MR, and in particular in the case of MR-Egger for two-sample MR,^36^^,^^38^ they could clearly be used as a sensitivity analysis to assess violation of the exclusion restriction criteria when several different treatment IVs are used in RCTs.

## Different directions of bias within the approaches being compared

Triangulation works best if within the set of approaches being compared there are some with differences in their expected directions of bias. These expectations will depend on the particular causal question being addressed; they cannot be predicted in general for each approach. However, the extent to which we can a priori determine this for a particular question is likely to vary depending both on the question and the approaches and data that are available to address it. Some approaches in a specific example might have several important sources of bias that have different directions (rather than one key source). In some triangulation examples, new sources of bias in some of the approaches might only become apparent once analysis begins.

Two of the methods (cross-context comparison and use of different controls) described in Table 1 have comparisons within them that are expected to result in bias in different directions, which makes them particularly useful in triangulation. In cross-context comparisons, results are compared between two or more different populations, or the same population in two or more different contexts (e.g. over different time periods), under the a priori assumption that confounding structures differ between the different contexts/populations.^21^ This assumption is based on prior knowledge but should be tested with observed confounders. The idea with this approach is that where there are concerns about the association being driven by residual confounding in one context/population, additional populations are sought in which the confounders of concern are unrelated to exposure or outcome (or related in the opposite direction) or do not vary in the additional populations. Thus, in the additional populations there would either be no confounding or confounding would affect the result in the opposite direction.

Using different controls in case control studies^22^ has similar underlying principles to those described above for cross-context studies, but here differences in exposure misclassification (rather than confounding structures) is the most common use of this approach.^22–24^ For example, exposure in bowel cancer cases might be compared with general population controls and with other cancer controls. The key source of bias with population controls is likely to be selective recall bias of the exposure (i.e. a tendency to over-report exposures that are thought to be risk factors in the population being studied), which in most cases would tend to bias results away from the null and towards the risk factor increasing cancer risk. With other (not bowel) cancer controls, selective recall bias is less likely, but it is plausible that the risk factor of interest (unknowingly) has a general carcinogenic effect or a specific effect on some of the ‘other cancers’ in the same direction as the effect in the bowel cancer cases. If that were true in analyses with other cancer controls, the bias would be towards the null.

In a third approach–negative control studies–we would expect an unbiased negative control study to be null and the ‘real’ study not-null if there is a true causal effect and the assumptions of this approach are met (see Table 1).^39^^,^^40^ In many negative control studies there will not be a simple dichotomy in which the ‘real’ study shows an effect but the negative control study is null (in the situation of a true causal effect). Rather, the negative control study may show a weaker (than the real study) association, suggesting some bias in the real study but, under the assumptions of a negative control study, there is also some causal effect. In that situation, the difference of the negative control results from the null can be used as an indicator of the extent of bias in the original study.^41^^,^^42^

Thus, including one or all of these three approaches (cross-context comparison, use of different controls in case control studies, and negative control studies) in a set of (additional) approaches using triangulation to address a causal question is advantageous. In addition, the greater the number of different approaches that are compared, the greater the likelihood that some approaches will have key sources of bias that would be expected to bias results in different directions. As we demonstrate in the illustrative examples, once approaches and data are decided on, it is important that key sources of bias and the likely overall direction of these for each approach are articulated and taken into account when comparing results in a triangulation framework.

## Duration and timing of exposure being assessed with different approaches

This issue is related to the criterion that the different approaches are addressing the same underlying question. However, we believe it is important to highlight this as a separate criterion because observational and RCT studies rarely make the exposure duration explicit as part of the research question. Although timing of exposure might be included as part of a research question–for example, many developmental origins questions are concerned with exposure during a particular (developmental) time period–this will not always be the case. For example, the hypothesis that the effect of hormone replacement therapy (HRT) on coronary heart disease (CHD) might differ depending on the timing of its use (in relation to menopausal status) was not raised until after results from RCTs were published.^47^^,^^48^ By emphasizing the importance of taking account of duration and timing of exposure in our criteria, triangulation might highlight the possibility of time-specific effects (i.e. generate a hypothesis), which can be further tested in a triangulation framework focused on the specific time-exposure question.

For many exposures, greater duration of exposure will result in greater risk. In relation to the different approaches that might be compared in a triangulation framework, RCTs will, in general, test exposure over a shorter duration than conventional approaches applied within prospective cohort studies, and MR studies will often test exposure duration across a much longer period of the life course (potentially from conception). However, it is important to acknowledge that assumptions made about how different approaches assess exposure duration or timing can be biased. In a triangulation framework this needs to be explored explicitly, as we do in the examples below. For example, whereas prospective cohort studies often will be studying exposures over longer time periods than RCTs, attenuation by errors (i.e. regression dilution bias) will influence prospective cohort studies more than trials in which you have reasonable estimates of the sustained differences in exposure generated by the intervention.

Timing of exposure, such as whether or not an exposure has an effect only during a critical period, or has different magnitudes of effect at different times in the life course (sensitive periods) are also important to consider.^49^^,^^50^ Some of the methods described in Table 1 might be less suitable for testing critical or sensitive-period exposure effects than others. For example, MR generally examines exposure across a large part of the life course and might be unable to distinguish critical or sensitive-period exposure effects. By contrast, a negative exposure control study is well suited to test such hypotheses where there are repeat measurements of the exposure and the exposure effect on outcome in the critical/sensitive period (‘real’ exposure) is compared with its effect on outcome outside this period (‘negative exposure’ control). This will be particularly the case, if the same methods to measure the exposure are used for all repeat assessments, as this will mean biases for the real and negative exposure controls will generally be the same. For example, we have compared the effect of gestational weight gain in the first trimester (where it mostly represents maternal fat deposition–the ‘real’ exposure) on offspring adiposity and cardio-metabolic outcomes with the same effects of gestational weight gain in later pregnancy (where the contribution from maternal fat deposition is relatively less and hence exposure to weight gain in these later periods is a negative control).^51^^,^^52^ Whether using a triangulation approach or not, if the timing of exposure is central to the research question then that needs to be specifically included in the hypotheses tested and the comparisons made.^49^^,^^50^

## Three illustrative examples of the use of triangulation in aetiological epidemiology

Each of the following examples are in areas where we have research interests. They do not cover all of the approaches described in Table 1, but across the three they illustrate several of the key concepts described above, including considering sources of bias, whether these are likely to be related across the approaches and their expected direction. With these examples we also illustrate the potential impact of duration and/or timing of exposure effect. These examples were specifically chosen to be somewhat ‘messy’ rather than proof of principle–so that together they illustrate the key principles, opportunities and challenges of triangulation. Full details of how we selected approaches and assessed the likely key sources of bias and directions of these are provided in Supplementary text available at *IJE* online. This Supplementary text illustrates the level of detail that we feel would be required for describing approaches used in a triangulation paper. It is not possible to include this text in the main paper as it is the equivalent of substantial parts of what might be three papers, and would hinder the flow and focus of this paper.

### Example 1: what is the causal cumulative effect of lower systolic blood pressure on CHD risk?

This first example is taken from a single publication by Ference *et al.*,^53^ in which the cumulative effect of lower systolic blood pressure (SBP) on CHD was assessed using MR and compared with results from a meta-analysis of prospective cohort studies^54^ and a meta-analysis of IV ratio estimates from RCTs of antihypertensives.^32^ Ference *et al.* did not use the word triangulation in their paper, but their comparison of different approaches potentially fits our proposed criteria. In order to explicitly use our triangulation framework, we have reviewed Ference *et al*.’s paper, and the two original papers from which the prospective cohort and RCT results were taken, and considered the likely key sources of bias and the direction of those for the three approaches. This includes some additional analyses of the MR approach, using data and methods that were not available at the time that Ference *et al.* published their paper (see Supplementary text available at *IJE* online).

**Figure 2.**
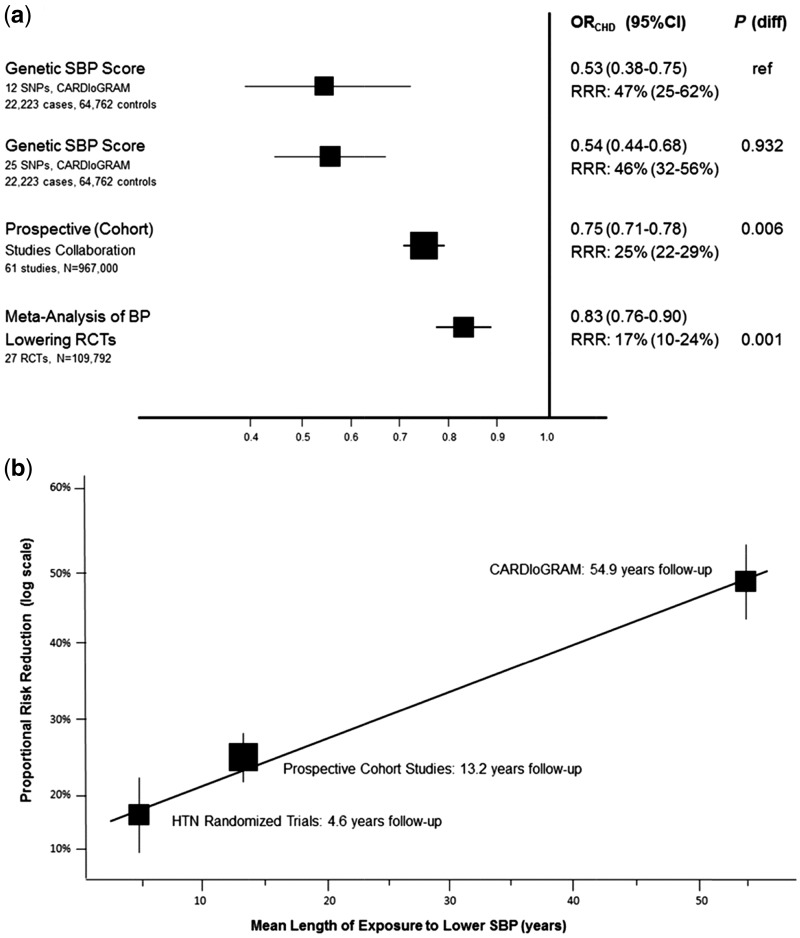
Triangulation of effect of systolic blood pressure on CHD risk from three approaches (RCT, multivariable regression and MR). Both graphs show the effect of exposure to 10 mmHg lower systolic blood pressure (SBP) on risk of coronary heart disease (CHD). In (a), squares represent the effect estimate for the association between 10 mmHg lower systolic blood pressure (SBP) and the risk of CHD; horizontal lines represent 95% confidence intervals (CI). The relative risk ratios (RRR) and their 95% CI are given for each approach on the righthand side of the graph. The *P*-values to the right of the RRR values (*P* diff) are testing the null hypothesis that results from the different approaches and are consistent with results from the first MR study (reference study). In (b), squares represent the proportional risk reduction (1−risk ratio) of CHD per 10 mmHg lower SBP plotted against the estimated mean length of exposure to 10 mmHg lower SBP; vertical lines represent 1 standard error (SE) above and below the point estimate of proportional risk reduction. Results are plotted against the estimated duration of exposure to lower SBP for each approach. Reproduced from reference 53 with permission.

Table 2 summarizes the key sources of potential bias and likely direction of these for each of the three approaches. These are discussed in more detail in web-based Supplementary text. Effect estimates from all three approaches point in the same direction of lower SBP causing reduced odds of CHD (Figure 2a), but the magnitudes of this effect vary, being greatest for the MR studies, intermediate for the prospective cohorts and least for the RCTs.^53^ It is possible that these differences in magnitude reflect different biases in the three approaches. However, the key sources of bias in the prospective cohort studies meta-analysis (possible residual confounding) and RCT (ignoring the impact of diastolic blood pressure (DBP) in addition to SBP on CHD) would likely exaggerate a positive effect, whereas our additional analyses suggest that the MR results likely have very little or no bias (Table 3; Figures S1–3, available at *IJE* online). Thus, our expected directions of key sources of bias would anticipate stronger (exaggerated) effects in the RCT and prospective cohort studies than in the MR approaches, whereas the results suggest the opposite. As Ference and colleagues suggest, the differences are more likely to be due to differences in the duration of exposure to lower SBP that each approach assesses. When this is taken into account. the three sets of results are broadly consistent with each other (Figure 2b). Thus triangulation suggests that lower SBP causally reduces CHD risk and the greater duration of exposure to lower SBP, the greater the CHD risk reduction.

**Table 2.** Different approaches used in triangulation to determine the effect of systolic blood pressure on CHD

**Approach**	**Brief description of approach**	**Key sources of bias and direction**^a^	**Duration of exposure assumption by Frerence *et al***^a^
Multivariable regression in prospective cohort studies^54^	Prospective Cohorts Collaboration: an individual participant meta-analysis of 958 074 adults (61 studies) aged 40–69 with no previous history of CVD. Exposure = SBP (in both Ference paper and original paper); outcome = fatal CHD	Residual confounding by adiposity and height, both of which **exaggerate** any true causal effect. Repeat SBP measurements in a large subgroup were used to adjust the association for regression dilution bias; the estimate of duration of exposure is therefore unlikely to be biased by this	From baseline SBP assessment to death or end of follow-up (mean 13.2 years)
IV of intermediate in RCTs^32^	Systematic review and meta-analyses of 25 RCTs including 109 797 participants with no clinical evidence of cardiovascular disease before randomization. Authors of the original paper calculated ratios of difference in log odds CHD ÷ difference in SBP/DBP by randomized group for each antihypertensive, and meta-analysed these. Exposure = SBP (in Ference (triangulation) paper but is actually the combined SBP and DPB effect in the original paper); outcome = fatal or non-fatal CHD	Ference *et al.* assumed the results represented a risk reduction in CHD for 10-mmHg lower SBP, whereas they were the risk reduction in CHD for a 10-mmHg lower SBP or 5-mmHg lower DBP. Thus the (assumed) SBP effect is **exaggerated** in comparison with its true effect	From randomization to end of follow-up (mean 4.6 years)
MR^53^	Two-sample MR. Genetic variants from the International Consortium for Blood Pressure genome-wide association study (ICBP) that had reached genome-wide levels of statistical significance were used.^77^ The association of each of those variants with CHD was extracted from the Coronary ARtery Disease Genome-Wide Replication And Meta-Analysis (CARDIoGRAM) consortium database (22 223 fatal or non-fatal CHD cases and 64 762 controls).^78^ The authors calculated ratios of difference in log odds CHD ÷ difference in SBP for each genetic variant, and meta-analysed them	We undertook a number of sensitivity analyses to explore the possibility of bias due to: (i) the fact that the ICBP results were for SBP adjusted for BMI; and (ii) violation of the exclusion restriction criteria (Supplementary text and Figures S1 to S3). On the basis of these we concluded these results were **unlikely to have major bias**	Whole of the participant’s life, and so mean age at end of follow-up or becoming a CHD case (54.9 years)

a In Supplementary text (available at *IJE* online) we provide full details of how we assessed a range of potential key sources of bias and their likely direction; here we describe the ones that we concluded were the key sources.

For this example, the three approaches were compared within one paper by Ference and colleagues,^53^ who undertook the MR study themselves but used results from published meta-analyses of prospective cohort and RCT studies. We have reviewed both Ference *et al*’s paper^53^ and the two papers^32^^,^^54^ that they took results from in preparing this table and the detailed Supplementary text.

**Table 3.** Different approaches used in triangulation to determine the effect of maternal circulating pregnancy glucose on birthweight

**Approach**	**Brief description of approach**	**Key sources of bias and direction**^a^	**Duration/timing of exposure**^a^
Multivariable regression (European)^55^	Multivariable regression in 6008 European mother-offspring pairs. Adjusted for offspring sex and gestational age	Residual confounding by maternal socioeconomic position, age, parity and adiposity, which would result in **exaggeration** of any true causal effect	Fasting glucose assessed at single time point (24–28 weeks of gestation). For cumulative effect we assumed this is from then until birth (i.e. the last 12–16 weeks)
Cross-cohort comparison^56^	Comparing multivariable regression between 750 Pakistani origin and 607 White British origin mother-offspring pairs	Because of differences in the associations of SEP with fasting glucose between the two populations [mean difference in glucose by maternal education in Pakistani 0.00 mmol/l (-0.03, 0.04) and in White British 0.04 (0.02, 0.06); by receipt of income support in Pakistani -0.25 (-0.44, -0.07) and in White British 0.10 (0.01, 0.21)], if an association in White British were due to SEP confounding, we would expect a **weaker association in Pakistani women**	Fasting glucose assessed at single time point (26–28 weeks of gestation). For cumulative effect we assumed this is from then until birth (i.e. last 12–14 weeks)
MR^55^	Use of a weighted allele score of genetic variants known to be robustly associated with fasting glucose as an IV in 11 493 European mother-offspring pairs	Methods, including sensitivity analyses, were undertaken in the paper to explore the possibility of bias due to: (i) weak instruments; and (ii) violation of the exclusion restriction criteria. On the basis of these we concluded that these results may be somewhat biased towards the null as a result of adjusting for offspring genetic variants (see Supplementary text)	Assumed this approach tests fasting glucose across the whole of pregnancy
IV of intermediate in RCT^57^	958 women with mild gestational diabetes mellitus randomized to dietary advice, glucose monitoring and insulin treatment if necessary or usual care. We calculated an IV ratio estimate (difference in birthweight by randomised group ÷ difference in glucose by randomised group)	Glucose was not monitored in the control arm and we brought the baseline value forward. Maternal fasting glucose levels increase in the second and third trimesters of pregnancy. As a result the denominator of the IV ratio estimate (i.e. difference in fasting glucose by randomized group) is likely to have been an underestimate of the true difference and the IV estimate of the effect of fasting glucose on birthweight an **exaggeration** of any real causal effect. Potential violation of the exclusion restriction criteria by dietary advice possibly result in changes to other risk factors that influence birthweight independently of glucose. The likely direction of effect of this bias is unclear (see Supplementary text). Overall, we decided that likely combined bias for this approach would be to exaggerate a positive effect of glucose on birth weight (Supplementary text)	Randomization and fasting glucose assessed at ∼ 29 weeks of gestation. For a cumulative effect we assume differences were present for the last 11 weeks of pregnancy

a In Supplementary text (available at *IJE* online) we provide full details of how we assessed a range of potential key sources of bias and their likely direction; here we describe the ones that we concluded were the key sources.

SEP, socioeconomic position.

### Example 2: what is the effect of maternal gestational circulating glucose levels on offspring birthweight?

We used evidence from studies that we were aware of, identified additional ones from literature searches and undertook some *de novo* analyses using data from studies that we have access to. This enabled us to compare results from multivariable regression in pregnancy cohorts,^55^ a cross-context comparison using data from the Born in Bradford (BiB) study,^56^ an MR study^55^ and IV analyses of an intermediate (fasting glucose) in an RCT.^57^Table 3 summarizes the approaches and potential key sources of bias, together with the likely direction of these. Supplementary text provides a more detailed discussion of these, available at *IJE* online.

**Figure 3.**
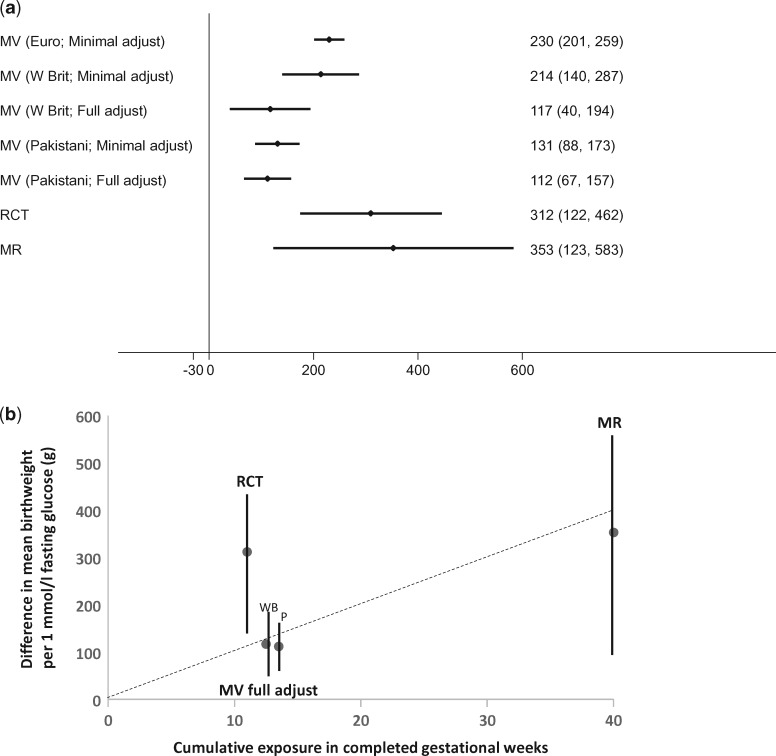
Results for triangulation across different approaches to determine the effect of maternal circulating pregnancy glucose on birthweight. a: Difference in mean birthweight (g) per 1 mmol/l greater fasting glucose. b: Difference in mean birthweight (g) per 1 mmol/l greater fasting glucose against the cumulative number of weeks of exposure (in completed gestational weeks) to 1 mmol/l greater fasting glucose. In (a), the effects are shown of 1 mmol/l maternal gestational fasting glucose on difference in mean birthweight in grams (g) from different approaches. MV, multivariable regression in prospective pregnancy cohorts; Euro, European-origin mother-offspring pairs; W Brit, White British mother-offspring pairs; minimal adjust, adjusted for infant sex and gestational age only; full adjust, fuller adjustment with additional adjustment for maternal age, BMI, parity, education and receipt of income support. In (b), the estimates are shown of the fuller adjusted MV analyses in White British (WB) and Pakistani (P) mother-offspring pairs, together with the IV analyses in the RCT and MR approaches, plotted against estimated length of cumulative exposure to fasting glucose for each approach in completed gestational weeks. The mean length of exposure in the MV of White British and Pakistani pairs is the same (13 weeks), but in order to visualize both they have been separated to 12.5 and 13.5 weeks. The regression line is forced through zero and shows that the RCT result appears to be an outlier (exaggerating the effect of glucose on birthweight).

Figure 3 shows the results from the different approaches of the effect of a 1-mmol/l greater maternal fasting glucose on birthweight. We found positive associations of maternal gestational fasting glucose with offspring birthweight in multivariable regression analyses pooled from European origin cohort studies with minimal adjustment for potential confounders.^55^ Evidence from our cross-context comparison suggested that there was residual confounding in the European multivariable approach, but that may not have fully explained a positive effect. With adjustment for gestational age and infant sex only, as in the European collaboration, White British women from BiB had the same magnitude of positive fasting glucose-birthweight association as in the European collaboration, whereas the Pakistani origin women [in whom confounding structures differed in ways that would mean we anticipated little confounding in this population (Table 3)] had a weaker positive association. Additional adjustment for maternal socioeconomic position, age, body mass index (BMI) and parity attenuated the associations in the White British BiB women towards the minimally adjusted results for the Pakistani women, whereas these adjustments in the Pakistani women did not notably alter its magnitude. Positive fasting glucose-birthweight effects were also seen in the IV of glucose as an intermediate in an RCT^57^ and IV MR studies.^55^

The magnitude of effect of glucose on birthweight from the IV RCT and IV MR studies were stronger (both > 300 g greater birthweight per 1 mmol/l greater fasting glucose) than that seen in the ‘fully’ adjusted multivariable analyses (∼ 115 g greater birthweight per 1 mmol/l) (Figure 3a). These differences could be due to different biases in the different approaches or differences in duration or timing of the exposure. We anticipated a priori that the RCT effects would be exaggerated due to the assumptions we had to make about change in glucose levels in the control arm, and also possibly due to violation of the exclusion restriction criteria (Table 3; and Supplementary text, available at *IJE* online), but thought that the MR results might be weakly biased towards the null because of adjustment for offspring genetic variants introducing a path from mother’s genetic IV to father’s genotype and to offspring outcome **(**Supplementary text, available at *IJE* online).

The differences in magnitude of effect between the different approaches might also reflect differences in duration or timing of exposure between the different approaches. The MR study will likely reflect mean differences in maternal glucose levels across the whole of pregnancy, whereas the multivariable regression analyses in the pregnancy cohorts are based on one (baseline) measure of fasting glucose taken between 26 and 28 weeks of gestation and thus reflect lower glucose during the last 12–14 weeks of gestation (assuming, as in Ference *et al.*,^53^ that exposure duration in a prospective cohort is from baseline assessment to end of follow-up). With a single measurement there might be some regression dilution bias of this association. Thus, the ∼ 115 g greater birthweight per 1 mmol/l greater fasting glucose effect estimate from this approach might be an underestimate of the cumulative effect of glucose levels across the last 12–14 months of pregnancy. Participants in the RCT were randomized at a median of 29 weeks of gestation, and those in the intervention arm had their glucose monitored from that time; that approach reflects the effect of lowered fasting glucose for the ∼ last 11 weeks of gestation. If duration or specific timing (or both) of exposure to glucose influenced its effect on fetal growth, these differences in exposure timing or duration between the studies might explain some of the differences in magnitude of effect.

It is possible that exposure to greater glucose in late pregnancy, when levels of glucose are higher and fetal growth and utilization of glucose is greatest, has a stronger effect on birthweight that glucose levels in earlier pregnancy (i.e. late pregnancy is a sensitive period). In that case, we would expect all three approaches to have a positive effect, but that findings from the multivariable regression and RCT approaches (which specifically test the exposure during that late period of pregnancy) to be stronger than those from MR (which tests the effect across the whole of pregnancy and may not identify individual differences in the rate of increase in glucose in late pregnancy). If there was no sensitive or critical period but greater duration of glucose exposure resulted in a greater effect on birthweight, we would expect to see weakest effects in the RCT, intermediate in the prospective cohorts and strongest in the MR. We observe none of these, rather we observe similar effects in the RCT and MR approaches and weaker effects in the multivariable regression analyses. When we plot the effect of each approach against estimated duration of exposure, as done by Ference *et al.*,^53^ the results provide some support for a cumulative duration effect, with a biased (exaggerated) effect from the IV analyses in the RCT (Figure 3b). However, for this example, we would suggest our triangulation effort highlights the need for additional evidence. More RCT evidence in which fasting glucose is monitored in both arms would be valuable, but given the lowered threshold of glucose for diagnosing gestational diabetes,^58^ together with effective interventions, it is unlikely that such RCTs would be conducted other than possibly in select groups such as obese women.^59^^,^^60^ Thus, additional multivariable regression analyses with repeat (continuous) glucose monitoring across a greater length of gestation in a number of different populations with differing confounding structures, and if possible within-sibship analyses, would be useful to compare with the existing MR and multivariable effect estimates.

### Example 3: What is the causal effect of having been breastfed on later body mass index?

We combined our own knowledge of studies examining this question with literature searches and identified a large systematic review and meta-analysis of prospective cohort studies,^61^ a cross-cohort comparison,^21^ three within-sibship studies (from two cohorts)^62–64^ and an RCT of breastfeeding promotion.^65^^,^^66^ We added a negative control study that we undertook for this paper, using data from the ALSPAC study.^67^^,^^66^Table 4 summarizes the approaches and the likely key sources of bias for the approaches used this example, with more detailed discussion of these in Supplementary text available at *IJE* online.

**Table 4.** Different approaches used in triangulation to determine the effect of having been breastfed on later body mass index

**Approach**	**Brief description of approach**	**Sources of and potential direction of bias**^a^
Multivariable regression^61^	Systematic review and meta-analysis of prospective cohort studies in participants largely of European origin, including up to 355 301 participants in different analyses. BMI assessed across ages from 1 to 70 years	Residual confounding by maternal SEP and BMI (the majority of studies did not adjust for these) which would produce an effect estimate that is an **exaggeration** of any real effect
Cross-context comparison^21^	Multivariable regression in a UK cohort (BMI assessed at mean age 9; *n* = 4852) compared with similar multivariable regression analyses pooled across five studies from low- and middle-income countries (LMIC). BMI was assessed mean age 15–41 years; *n* = 10 912	It was demonstrated that SEP did not relate to breastfeeding in the LMICs or was in the opposite direction (more affluent and educated women being less likely to breastfeed) to that seen in the UK (breastfeeding more common in the more affluent and educated women). If an association in UK participants was due to SEP confounding, we would expect a **null or opposite direction association in those form LMICs**
Within-sibship comparisons^62–64^	Three studies were identified which examined associations of being breastfed with BMI. Two of these used the same data from the US National Longitudinal Study of Adolescent Health (Add Health) and had similar results;^62^^,^^63^ we present results from the study that focused on mean BMI (2734 sib-pairs; ages 12–18).^62^ The third study was also US-based and included 488 sib-pairs whose BMI was assessed at ages 9–19 years^64^	In both studies breastfeeding was retrospectively reported by mothers when the children were adolescents, and misclassification is potentially the key source of bias here, which would be likely to result in an **attenuated** estimate of any true causal effect. Neither study had adequate statistical power to detect a difference between the effect estimates from the within-sibship analyses and that of unrelated participants in the cohorts
RCT^65^^,^^66^	17 046 Belarusian women with healthy singleton births were randomized to a breastfeeding promotion intervention or usual care. The intervention resulted in marked differences in ever breastfeeding, duration of breastfeeding and whether breastfeeding was exclusive. An intention- to-treat analysis was used to assess the impact of these differences on BMI at age 6.5 (*n* = 13 879) and on BMI and fat mass index (FMI) at age 11.5 (*n* = 13 866).	Because the intervention affected multiple aspects of breastfeeding, it was not possible to do a formal IV analysis, but the intention-to-treat analyses would have similar assumptions to an IV analysis to test an intermediate. There would be violation of the exclusion restriction criteria if the intervention, in addition to influencing breastfeeding, also affected the mothers, such that they had a tendency to a healthier lifestyle more generally, including encouraging their child to have a healthier diet and be more active postnatally. That violation would result in an effect estimate that was an **exaggeration** of any true causal benefit of breastfeeding
Negative control study^67^^,^^68^	We tried to identify outcomes in the ALSPAC cohort^67^^,^^68^ that would be affected by confounders that are important in the relation of breastfeeding to later offspring outcomes but for which a biological/causal effect of breastfeeding was unlikely (home invasion by mice and by pigeons)	If the negative control outcomes are biologically influenced by the exposure, the interpretation of this approach would be biased. However, we consider it unlikely that house infestation by pigeons or mice would be affect being breast-fed, other than through confounding or other sources of bias. We explored associations of observed confounders with these outcomes and these verified that these were appropriate negative controls (Table S3)

a In Supplementary text (available at *IJE* online) we provide full details of how we assessed a range of potential key sources of bias; here we describe the ones that we concluded were the key sources. As the length of exposure (to breastfeeding) varied across the studies or was not measured in some, we have not commented on duration of exposure; timing will be similar in all approaches.

Results are tabulated (Table S2, available at *IJE* online) rather than shown in graphs as in the previous two examples, because of differences between studies in how breastfeeding was assessed (e.g. never versus ever in some studies, duration in others). There was an inverse association of ever being exclusively breastfed, and of being exclusively breastfed for at least 8 months, with mean BMI in a meta-analysis of prospective cohort studies with minimal (age and gender) adjustment, but results were attenuated with adjustment for maternal BMI, smoking and socioeconomic position and there was evidence of publication bias.^61^ Cross-context comparisons between a UK cohort and pooling of data from five low- or middle-income countries (LMIC) showed an inverse association of duration of breastfeeding with BMI in the UK cohort but not in the LMIC cohorts.^21^ In the LMICs, socioeconomic position was not notably related to breastfeeding or related to it in the opposite direction to that seen in the UK cohort. The largest^62^^,^^63^ of the two within-sibship comparisons suggested that any associations observed between unrelated individuals were explained by shared familial confounding, though the within-sibship analyses in both studies were imprecisely estimated and not reliably different from the results of analyses between unrelated individuals.^62–64^ The large RCT of a breastfeeding intervention that resulted in marked differences in breastfeeding practice showed no effect of randomization on BMI at mean age 6.5 years or on BMI or fat mass index at age 11.5 years.^65^^,^^66^ These null results could not plausibly be explained by violation of the exclusion restriction criteria through long-term effects of the intervention on other health-promoting activity, as that would be anticipated to result in a reduced BMI in those randomized to intervention (Table 4; and Supplementary text, available at *IJE* online).

Last, our negative outcome control study suggested that the observed inverse association of having been breastfed with childhood obesity was likely due to residual confounding (Table S3, available at *IJE* online). We describe in Supplementary text how we selected valid negative control outcomes and the assumptions of this approach. Our negative controls were binary–home invasion by mice and home invasion by pigeons. Therefore we compared associations between breastfeeding and the control outcomes with the association between breastfeeding and child obesity (as a binary outcome). Based on our a priori assumptions regarding how confounders would related to these negative control outcomes (Supplementary text) and the testing of these with observed confounders (Table S3), we anticipated that if the ‘real’ inverse association of being breastfed with obesity was due to confounding that was mimicked in the negative controls, we would see a positive association between home invasion of mice and obesity and an inverse association between invasion of pigeons and obesity (Supplementary text). We found that having been breastfed was inversely associated with obesity at age 7, but it was also inversely associated (with the same magnitude) with parental report of home invasion by pigeons and positively associated (stronger magnitude) with report of invasion by mice (Table S3).

Thus, with the exception of the within-sibling analyses, which were too small to provide reliable estimates, all of the approaches in this example point to no meaningful causal association between having been breastfed and later BMI.

## Concluding remarks

The aim of this paper is to raise the profile of integrating evidence from different epidemiological approaches in a triangulation framework to address aetiological questions. We are not claiming that this is a new approach that has not been previously suggested or used. Our definition and criteria for triangulation are similar to some other approaches to causality, including Hill’s concept of consistency^16^ and work by Susser,^69^^,^^70^ Morris^71^ and more recently Shipley,^72^ all of which represent responses to Duhem and Quine’s contention that no hypothesis is tested in isolation and always involves auxiliary hypotheses and information.^73^ This paper differs from these works in its attempts to define triangulation, establish criteria for its use in aetiological epidemiology and demonstrate its use with examples that illustrate some of its potential and challenges. The process for conducting International Agency for Research on Cancer (IARC) monographs also has some similarities to triangulation, in that the aim is to identify and review ‘all relevant papers on cancer in humans and experimental animals …’^74^ By necessity this review process will involve consideration of the different sources of bias in each approach used in the identified papers. For example, a recent monograph on the causal effect of fatness on cancer compares (human) evidence from multivariable regression in observational studies, including exploring consistency of findings across a number of different populations that might potentially have different confounding structures, and MR studies where these were available, and concluded that there is sufficient evidence to support a causal preventive effect of lack of excess body fat on several cancers.^75^ However, these monographs are not explicit about using a triangulation approach. They aim to identify all published evidence but do not specifically seek to identify approaches with different key sources of bias or make inferences on the basis of explicitly comparing studies with different biases.

In this paper we have not considered how to pool data in a triangulation framework in order to produce a quantified causal effect estimate; neither have we considered effect modification (beyond modification by age when considering timing of exposure). Pooling of data (as in meta-analyses) assumes that all studies give unbiased estimates of the same underlying causal effect, and can be pooled to give one estimate. Since triangulation involves integration of data from very different approaches with the explicit intention of having expected biases in (at least) some of the approaches, simple pooling would not be appropriate. Methods could be developed to apply to triangulation to give plausible bounds of causal effect, including taking account of duration and timing of effect, and possibly exploring whether there are different bounds of causal effects in different subgroups (suggesting effect modification), but that methodological development is beyond the scope of this paper.

Some of the approaches that we describe could be considered a form of triangulation within themselves. Cross-context comparisons with many different populations each with different confounding structures, RCTs of intermediates that use multiple different treatments (for example different classes of antihypertensives to test the effect of blood pressure on CHD^31^) and MR with multiple genetic instruments that relate to the exposure of interest through different mechanisms, might be considered in this way. However, comparisons across multiple populations or multiple IVs are really a way of testing the extent to which the key sources of bias within those approaches are minimized, rather than triangulation, in which different approaches with different (and unrelated) key sources of bias are compared.

Increased use of triangulation in aetiological epidemiology is likely to require multidisciplinary collaboration, more extensive data sharing and additional resources in comparison with applying just one (conventional) approach. We would argue that the potential gain in aetiological understanding is worth the extra effort. Any additional cost should be weighed against the cost of developing and trialling interventions based on unsound evidence, which contributes to the large number of treatment or preventive targets that are evaluated and ultimately turn out to be ineffective at great cost to human participants, industry and society.

Considerable advances could be made within the triangulation framework, at modest cost but with greater access to research data. As more observational studies have genetic data, it will be increasingly possible to compare conventional multivariable regression approaches, MR and possibly negative control (exposure or outcome) studies, within the same datasets. Cross-context comparisons would be enhanced by greater sharing of research data from cohorts across the globe. The application of IV analyses to test causal effects of intermediates in RCTs, or the use of RCTs to test effects of the primary intervention on a range of outcomes, would be enhanced by greater sharing of data.

Greater data access is increasingly promoted and there are some examples of good practice. For example, individual participant data from the large UK Biobank (which currently has genotypic, phenotypic and clinical data in up to 500 000 adults^76^) and the Avon Longitudinal Study of Parents and Children (with very detailed data across three generations across their life course^67^^,^^68^) are available to researchers globally at modest cost (the amount required to prepare datasets and data information). The aim of *IJE* cohort profiles is to increase the ease of data sharing globally, with all profiles having to include a section on ‘How can I get hold of the data?’. Many genome-wide association consortia have now made their aggregate results fully available (i.e. all genetic associations for up to several million genetic variants with an ever-increasing number of outcomes from molecular to behavioural phenotypes, irrespective of *P*-values), with massive potential for these to increase aetiological understanding, including mechanistic insights, through two-sample MR.^38^ However, we feel these examples of data sharing should be more widespread in the community with funders, journal editors and governing bodies ensuring this greater data access.

In conclusion, we believe that triangulation has considerable potential to improve causal inference in aetiological epidemiology, which will be enhanced with increased data access and multidisciplinary collaborative work. As with other areas of data integration and development of novel methods for improving causal inference, attempts to use this approach are likely to yield further developments including analytical methods that could improve how to quantitatively combine data to obtain bounds of likely magnitudes of causal effect.

## Supplementary Data

Supplementary data are available at *IJE* online.

## Funding

D.A.L, K.T and G.DS work in a unit that receives funds from the University of Bristol and the UK Medical Research Council (MC_UU_12013/1, MC_UU_12013/5 and MC_UU_12013/9).The research leading to this paper has received funding from the European Research Council under the European Union’s Seventh Framework Programme (FP7/2007-2013) / ERC grant agreement no669545. D.A.L is a National Institute of Health Research Senior Investigator (NF-SI-0611-10196).

**Conflict of interest:** The authors declare that they have no conflict of interest.

Key messages

- Triangulation involves addressing a causal question by integrating results from several different approaches that have different and unrelated key sources of potential bias.
- We propose a minimum set of criteria for the use of triangulation in aetiological epidemiology: (i) results from at least two, but ideally more, different approaches, with differing and unrelated key sources of potential biases, are compared; (ii) the different approaches address the same underlying causal question; (iii) related to (ii), for each approach the duration and timing of exposure that it assesses is taken into account when comparing results; (iv) for each approach, the key sources of bias are explicitly acknowledged when comparing results; (v) for each approach, the expected direction of all key sources of potential bias are made explicit where this is feasible, and ideally within the set of approaches being compared there are approaches with potential biases that are in opposite directions.
- Where results from two or more approaches fulfilling these criteria point to the same answer, this strengthens causal inference. Pointing to the same conclusion does not mean that the results are statistically consistent and could be pooled; currently triangulation will mostly provide a qualitative assessment of the strength of evidence regarding causality.
- Where results point to different causal answers, understanding the key sources of bias can help direct researchers to what further research is needed to answer the causal question.

## Supplementary Material

Supplementary Data
