# Supplementary material for: Triangulation in aetiological epidemiology
Source: Int J Epidemiol. 2017 Jan 20;45(6):1866–86. doi: 10.1093/ije/dyw314 (PMC5841843; doi:10.1093/ije/dyw314)
Supplement: Supplementary Data [file dyw314_supp.zip › dyw314-suppl_data/DONOT USE THISije-2016-05-0586-File002.docx]

**SUPPLEMENTARY MATERIAL FOR:**

**Triangulation in aetiological epidemiology.**

Debbie A Lawlor^1,2^, Kate Tilling^1,2^, George Davey Smith^1,2^.

^1^ MRC Integrative Epidemiology Unit at the University of Bristol, UK

^2^ School of Social and Community Medicine, University of Bristol, UK

**Corresponding Author**

DA Lawlor MRC IEU, University of Bristol, Oakfield House, Oakfield Grove, Bristol, BS8 2BN, UK

Email: [d.a.lawlor@bristol.ac.uk](mailto:d.a.lawlor@bristol.ac.uk)

|  |  | Page |
| --- | --- | --- |
| Supplementary Text (S-text) | Detailed descriptions of methods and likely sources of bias and their directions for the three illustrative triangulation examples | 2-21 |
| Table s1 | Examples of using different epidemiological approaches to answer aetiological questions | 22-28 |
| Table s2 | Results of studies used in triangulation to examine the effect of having been breastfed on later BMI | 29-30 |
| Table s3 | Negative control outcome study of effect of having been breastfed on BMI: using home infestation by mice and pigeons as negative controls for overweight/obesity. | 31 |
| Figure s1 | Sensitivity analysis to explore pleiotropy resulting in violation of the exclusion restriction criteria in the MR approach used in the illustrative example 1 | 32-33 |
| Figure s2 | Exploration of outlying genetic variant in the 2-sample MR used in illustrative example 1: Cooks distance and studentized residuals | 34-35 |
| Figure s3 | Sensitivity analyses of 2-sample MR used in illustrative example 1: Results with outlier SNP removed | 36-37 |
| Supplementary references |  | 38-43 |

**Supplementary text (s-text):**

**Description and assumptions of the MR-Egger and Weighted median methods for testing violation of the exclusion restriction criteria in Mendelian randomization**

*MR- Egger*

The MR-Egger method was developed to specifically test for genuine (also known as horizontal) pleiotropy and correct for this in MR analyses using aggregate data from multiple instruments,^1^ as done in two-sample MR.^2^ It uses a weighted linear regression of each of the genetic IV–outcome coefficients θ_j_ on the genetic IV–exposure coefficients δ_j_: θ_j_ = β_0E_ + β_E_*δ_j_, in which all the δ_j_ associations are orientated to be positive, and the weights in the regression are the inverse-variances of the gene–outcome associations ($\sigma_{Y_{j}}^{-2})$. **If** the intercept from this regression model was constrained to zero, the MR-Egger slope estimate β_E_ would be the same as the β from the more conventional inverse variance weighted pooling of estimates from each genetic IV. However, in MR-Egger the intercept is not constrained to zero and therefore the intercept (β_0E_) provides an estimate of the average pleiotropic effect across all of the genetic variants. This is because if there is no genuine pleiotropy (i.e. no violation of the exclusion restriction criteria) then there should be no change in outcome when the gene IV-exposure association is zero (i.e. intercept should be zero). If the intercept is not zero then its value is the difference in outcome that is not due directly to the risk factor that the gene IV is instrumenting for and this when an intercept term that differs from zero it suggests genuine (horizontal) pleiotropy. The MR-Egger slope then provides a causal estimate of the risk factor of interest having adjusted for this violation of the exclusion restriction criteria.

The MR-Egger method provides consistent estimates (from the MR-Egger slope) for the true casual effect even if all genetic variants are invalid due to horizontal pleiotropy (i.e violation of the exclusion restriction criteria), but requires an additional (to the standard IV assumptions) assumption known as the InSIDE (instrument strength independent of direct effect) assumption. This assumes that pleiotropic effects on the outcome are direct and not via confounder(s). If the pleiotropic effects of genetic variants are all via a single confounder, they will be correlated with instrument strength, and thus the InSIDE assumption will be violated.

*Weighted medians*

A simple (without weights) median method is obtained from the median of the ratio estimates from each genetic variant used as an IV.^3^ Specifically, let β_j_ denotes the j^th^ ordered ratio estimate and assume that all βs (of the ratio estimate) are arranged from smallest to largest. If the total number of genetic variants is odd (i.e. j = 2k + 1, where k is one of the whole numbers), the simple median estimator is the middle ratio estimate β_k+1_. If it is even (i.e. j = 2k), the median is interpolated between the two middle estimates ((β_k_+β_k+1_)/2). The weighted median estimator is and extension of this method that is more efficient that the method without weights. It is the median of a distribution having estimate β_j_ as its P_j_ = 100(S_j_ − W_j_/2)^th^ percentile, where P is the percentile for the j^th^ ordered ratio estimate, w_j_ is the weight given to the j^th^ ordered ratio estimate, proportional to the inverse of the IV variance, and S_j_ is the sum of weights up to and including the weight of the j^th^ ordered ratio estimates, calculated using the following equation.

$Sj=\sum_{K=1}^{j} W_{k}$

This method (in addition to the standard IV assumptions) assumes that:

1. no more than 50% of the genetic IVs are invalid
2. no single IV contributes more than 50% of the weight.

**Detailed descriptions of methods and likely sources of bias and their directions for the three illustrative triangulation examples**

**Illustrative example 1: the causal effect of systolic blood pressure (SBP) on coronary heart diseases (CHD)**

The first example uses data from a published paper by Ference and colleagues^4^ that compares the effect of SBP on CHD from three approaches – multivariable regression in prospective cohort studies, an instrumental variable (IV) analysis applied to RCT data and a Mendelian randomization (MR) study. Table 2 in the main paper summarises the approaches used and their likely key sources of bias, below we describe these in more detail, including additional analyses that we undertook to explore the possible sources and directions of bias.

***Multivariable regression in prospective cohorts*** Summary results from the Prospective Cohorts Collaboration were used (inverted so that the results were presented as the risk reduction in CHD for a 10mmHg reduction in SBP).^5^ This was an individual participant meta-analysis of data from 958,074 adults (61 studies) who were aged 40-69 with no previous history of cardiovascular disease. The study examined associations of SBP and DBP with fatal cardiovascular outcomes; Ference et al. focused solely on the results for the association of SBP with fatal CHD. Cause of death was obtained from death certificates, with validation by comparison with medical records and autopsy results in some studies. As the authors of the original paper noted, misclassification of outcome was unlikely to have been a key source of bias.^5^ Some studies used participant-reported SBP but the authors stated that results did not notably differ between those studies and studies with measured SBP. Individual studies varied in how they dealt with participants who were taking antihypertensive medication, with most adding a constant of either 10mmHg or 15mmHg; again the authors reported that results did not differ by how use of treatment was taken into account. The results presented were adjusted for age, sex and specific study only, though the authors wrote that additional adjustment for total cholesterol, HDLc, non-HDLc, diabetes, weight, alcohol and smoking did not materially alter the results.^5^ The potential confounding effect of BMI, or other measurements of adiposity, which is strongly positively associated with SBP and CHD, was not assessed, though in part will have been captured by weight, diabetes and the lipid adjustments. Similarly height, which is inversely associated with SBP and CHD, could have resulted in residual bias that would exaggerate the positive effect of SBP on CHD.

In comparing results across the three approaches Ference et al. made assumptions about the duration of exposure to lower SBP that cannot be fully tested.^4^ For the prospective cohort studies, exposure duration was said to be the length of follow-up and was given as 13.2 years.^4^ Length of follow-up in the original publication was not given for each individual cohort but it was stated that in the pooled data there was a mean of 12-years to death. Using the length of follow-up as exposure duration assumes that the ranking of participants according to their SBP measurement at baseline stays similar from then up to the end of follow-up. Without repeat measurements, short-term biological variability and longer-term changes to lifestyle and treatment for hypertension could bias any true relationship between SBP and CHD, with the direction expected to be towards the null. However, in this study data on 286,000 repeat SBP measurements from different studies covering the full length of follow-up allowed time-varying regression dilution bias to be taken into account, meaning that these changes over time are accounted for and regression dilution bias (attenuation by time-varying error) is unlikely to have biased the results.^5^

Thus, for this approach we consider that residual confounding by adiposity and height is the key source of bias and that this would result in a causal estimate that was an ***exaggeration*** of any true causal positive effect.

***Instrumental variable analysis applied to randomised controlled trials to test the effect of an intermediate (SBP) on CHD.*** Ference et al, used results from a systematic review and meta-analyses of 25 RCTs including 109,797 participants with no clinical evidence of cardiovascular disease prior to randomization and that aimed to produce a marked difference in BP lowering between the randomised groups.^6^ The RCTs compared active treatment to a specific target BP reduction (mostly pursued by increasing the dose of a single drug, occasionally with addition of another class of drug) to a placebo or standard care control group. Over 85% of the RCTs compared the intervention medication to a placebo, including studies that added a new treatment or placebo to an existing older antihypertensive in both arms (e.g. the intervention arm might include treatment with an ACE-inhibiter in addition to a standard dose of beta-blocker and the control arm a placebo plus the same dose of the same beta-blocker). BP lowering targets were also applied to the control arm in most RCTs, with increases in placebo prescribed initially but treatment with antihypertensives if participants reached a pre-specified high BP level. In most of the RCTs, the practitioner monitoring BP and altering treatment or placebo dose was different from the person evaluating the outcomes, who remained blind to allocation group. The outcome of these trials was fatal or non-fatal CHD. The causal effect of BP on CHD was estimated by a ratio estimator (i.e. log odds of CHD by randomised trial arm ÷ mean difference in BP per randomised arm) for each RCT of each antihypertensive, scaling these on the effect per 10mmHg lower SBP or 5mmHg lower DBP and then pooling these results in a meta-analysis. Although not described as such by the authors, this is an IV analysis of intermediates (SBP and DBP) of the randomised intervention (antihypertensives) and a key assumption is that randomisation to antihypertensives does not influence CHD through any path other than via their effect on BP (i.e. there is no violation of the exclusion restriction criteria as discussed in **Figure 1** of the main paper).

RCTs were in participants with hypertension or high BP (average SBP in the control groups ranged from 132mmHg to 186mmHg across the 27 trials). However, observational evidence suggests that the relative risk reduction in CHD is similar across initial levels at least down to 115mmHg for SBP.^5^ Thus, the select population in these RCTs is unlikely to have been a major source of bias as an estimate of causal effect in the general population.

The effect of lowering BP by a given amount on CHD was virtually identical for each different class of antihypertensive. As the authors of the original meta-analyses noted, this suggests that the effect of the drugs on CHD was largely due to their effect on BP and the causal effect estimate of BP reduction on CHD was not biased by “*pleiotropy*” (i.e. the exclusion restriction criteria was not violated).^6^ However, in their comparison of the meta-analysis of RCTs to their MR results and the prospective cohort results, Ference et al. report the BP lowering effect as if it were only for a 10mmHg reduction in SBP, whereas antihypertensives reduce both SBP and diastolic BP (DBP) and the results of the IV analyses applied to the RCTs in the actual paper is of the combined effect of an average 10mmHg reduction in SBP and 5mmHg reduction in DBP.^6^ This means that the effect that is being claimed as for a reduction in SBP only is exaggerated (i.e. bias it away from the null).

The length of follow-up of the 27 RCTs ranged from 1.1 to 8.4 years and in comparing the three approaches Ference et al. assumed an average of 4.6 years of follow-up. On average 25% of participants in the active treatment arms of the RCTs did not adhere to the treatment. The authors of the original meta-analysis correctly pointed out that the non-adherence would result in an underestimate of the effect of the antihypertensive treatments on CHD in intention to treat analysis, but would not bias the IV effect estimate of a given reduction in BP on CHD, because that effect was based on the observed BP difference between groups, which by definition would take account of whether or not the participants took the treatment.^6,7^ However, there was no report of adherence in the control groups; nor was information on loss to follow-up in the two arms and how that was dealt with in the intention to treat analyses reported. If adherence and loss to follow-up did not differ between the two arms of the RCTs by factors that impact on the treatment effect of antihypertensives on BP and also on CHD risk, then as the authors explain for non-adherence in the treatment arm there should be no bias of the effect estimate of BP on CHD. Without further information it is impossible to tell what the magnitude or direction of such bias might be.

Overall, we considered that for this approach the key source of bias is ignoring the combined SBP and DBP effects of antihypertension which would exaggerate the true effect of SBP on CHD ***(***i.e. ***exaggerate*** the hypothesized positive association***)***.

***MR*** Ference et al. used two-sample MR from publicly available summary genome wide association study results.^2^ They identified all genetic variants from the International Consortium for Blood Pressure genome-wide association study (ICBP) that had reached genome-wide levels of statistical significance in their associations with SBP.^8^ They then went to the publicly available Coronary ARtery Disease Genome-Wide Replication And Meta-Analysis (CARDIoGRAM) consortium database,^9^ of 22 223 CHD cases and 64,762 controls, and looked across the complete summary genome wide associations that are presented there as the log odds of CHD for each of the ICBP genome-wide significant variants. The effect estimate of each SBP variant on log odds of CHD from CARDIoGRAM was divided by the effect estimate of difference in mean SBP on each SNP from ICBP and those ratios pooled using inverse variance weighted (IVW) random effects meta-analysis. The resulting pooled estimate, which was in terms of log odds for CHD per 1mmHg lower SBP, was then converted to an odds ratio per 10mmHg lower SBP for comparison with the other two approaches. Two IV estimates were examined, one using 12 of the original 26 ICBP identified SBP variants and one using 25 of those variants (one of the ICPB SBP related SNPs was not available in the CARDIoGRAM consortium database).

The genetic variants that were used as IVs were robustly associated with SBP in ICBP, but all of the GWAS analyses in ICBP were adjusted for BMI. Thus, these genetic variants are actually IVs for the effect of SBP adjusted for BMI on CHD. Aschard et al. have shown that in GWAS that adjust for other heritable traits like BMI the resulting GWAS associations may be biased.^10^ In this case we would expect the bias to be away from the null in comparison to the true effect of genetic variants on SBP, but we explored this in an independent dataset (see below).^10^ If the genetic variant IV-SPB association is positively biased then the MR estimate of the effect of SBP on CHD would be to bias it towards the null because the denominator of the ratio (the gene-SBP association) which was taken from ICBP is exaggerated in comparison to the true gene-SBP association due to the BMI adjustment.

We have previously explored the extent to which this is likely to have biased MR effect estimates by using independent data from UK biobank and comparing the association of each SBP related SNP from ICBP with SBP with and without adjustment for BMI.^11^ These results show that as expected adjustment for BMI increases the magnitude of the associations for most of the SNPs but the actual magnitude of the difference is small, with the mean difference in regression coefficients comparing with to without BMI adjustment being 0.001mmHg (95% limits of agreement: -0.003 to 0.005). Thus, any bias from BMI adjustment of the gene-SBP association is likely to be minimal.

The MR results might be biased by genuine pleiotropy (violation of the exclusion restriction criteria; **Figure 1** in the main paper) if the SNPs related to SBP are related to other risk factors for CHD independently of SBP.^12^ The direction of effect of this would depend upon the pleiotropic factors. The authors argue that by using multiple SNPs and finding little heterogeneity between the IV effects of each separate variant, that it is unlikely that genuine pleiotropy has biased their results. However, they do not report heterogeneity statistics and the results across all 25 SNPs do vary from a log odds of CHD per 1mmHg lower SBP of -0.15mmHg (95%CI: -0.22, -0.08) to +0.05mmHg (95%CI: 0.03, 0.07). Since publication of the paper by Ference and colleagues several methods for exploring and potentially accounting for pleitropy have been developed,^1,3^ and in Supplementary **Figure s1** we show the results of applying these as sensitivity analyses to the summary data from ICBP and CARDIoGRAM that Ference and colleagues used. The results presented in **Figure s1** are for a 1mmHg increase in SBP because this is how these data are presented in ICBP. The IVW and weighted median meta-analysis results were similar to each other (**Figure s1a**), and if we scale the IVW results to the same as in the Ference paper (i.e. per 10mmHg lower SBP) they confirm those findings. However, we found evidence of heterogeneity between the IV ratio estimates of the 25 SNPs (p = 1.03 × 10^-5^) and the MR-Egger analysis suggested that there was violation of the exclusion restriction criteria and, that when this was taken into account, SBP did not appear to have a causal effect on CHD (**Figure s1a and s1b**). These results were largely influenced by one of the SBP genetic variants, rs17249754 in the ATPase plasma membrane Ca2+ transporting 1 (*ATP2B1*) gene, which is involved in intracellular calcium homeostasis, and is strongly associated with higher SBP but in the CARDIoGRAM consortium is associated with reduced CHD. Further tests (Cook’s distance and studentized residuals) confirmed that this variant was an outlier (**Figure s2**). When it was removed all three of the IVW, weighted median and MR-Egger meta-analysis results were consistent with each other and with those of Ference et al (**Figure s3**). The mean F-test with this SNP removed remained high at 51, suggesting there should not be a problem here with weak instrument bias. Any weak instrument bias in this two-sample MR would bias findings towards the null.^2^ Thus, our further sensitivity analyses suggest that the ***MR results are unlikely to have suffered from major bias***.

Given the different nature and sources of (i) residual confounding in the prospective cohort studies and (ii) ignoring the impact of antihypertensives on diastolic (as well as systolic) BP in the RCTs it is very unlikely that these two sources of bias would be related with each other. However, the likely direction of bias for these two is the same. If we are wrong in our assumption that the MR results are largely unbiased then it is possible that a positive effect in all three is due to an “exaggerating” bias. Given the exploration of bias in the MR that we have undertaken we think this is unlikely. Therefore in this example three approaches are compared, one that we think is likely to be unbiased (reflecting the true causal effect) and two in which unrelated key sources of bias would potentially exaggerate that effect. The results of the three approaches should be compared with that in mind (see main paper).

**Illustrative example 2: What is the effect of maternal gestational circulating glucose levels on offspring birthweight?**

Higher maternal circulating glucose crosses the placenta by facilitated diffusion, so that each increment greater maternal glucose is related to a higher delivery of glucose to the fetus.^13^ Maternal insulin does not cross the placenta and therefore greater maternal circulating glucose results in increased fetal pancreatic secretion of insulin which acts as a growth hormone.^13^ It would therefore be anticipated that greater maternal glucose results in greater birthweight. This is supported by a large body of observational epidemiology showing a positive association of gestational diabetes with large for gestational age birthweight or macrosomia,^13^ and also from the results of RCTs showing lower birthweight with treatment for gestational diabetes.^14^ Whether there is an effect across the range of fasting glucose is less clear.

We used evidence from studies that we were aware of, identified additional ones from literature searches and undertook some *de novo* analyses using data from studies that we have access to. This enabled us to compare results from multivariable regression in pregnancy cohorts,^11^ a cross-context comparison undertaken for this paper using data from the Born in Bradford (BiB) study,^15^ an MR study,^11^ and IV analyses of an intermediate (fasting glucose) undertaken on results from an RCT^16^. **Table 3** in the main paper summarises the approaches, potential key sources of bias, together with the likely direction of these. Below we provide more detail of the methods that we used and their likely sources and direction of bias.

The ***multivariable regression analysis*** was from meta-analysis of several prospective birth cohort studies of singleton pregnancies in European-origin women.^11^ It was undertaken alongside an MR analysis in the same cohorts and because of limited data on potential confounders in the collaboration was adjusted only for gestational age and infant sex. Thus, residual confounding by potential confounding factors (maternal age, socioeconomic position, parity and BMI) mean that the association is likely to be an ***exaggeration of the true effect***. The ***cross-context comparison***, undertaken for this paper, using data on a random subset of the Born in Bradford (BiB) cohort in which approximately half of the participants are UK resident women of Pakistani ethnic origin and half British (European) origin,^15^ allowed us to explore the extent to which that residual confounding might have biased the earlier multivariable results from the European collaboration. We were able to adjust for a range of relevant confounders in both groups and compare findings between the two groups. Given their relationships to maternal circulating glucose levels and birthweight, we considered maternal socioeconomic position, age, parity and BMI to be important confounders that would exaggerate the effect of maternal fasting glucose on birthweight, and we tested the associations of each of these potential confounders in both ethnic groups. Maternal age, BMI and parity had similar relationships to fasting glucose in both ethnic groups, but there were notable differences in the relation of socioeconomic position to fasting glucose between the two ethnic groups (see **Table 3 i**n the main paper), which mean that if the positive association in Europeans was notably exaggerated by socioeconomic position confounding then we might anticipate no effect or little effect in the Pakistani women.

We searched the literature to find any ***RCT***s of an intervention aimed at changing maternal circulating glucose in which the effect of the intervention on glucose levels, as well as on birthweight, were reported. We only found one study that fulfilled these criteria.^16^ In that RCT women with ‘mild gestational diabetes’ were randomized at a median 29 weeks of gestation to (i) dietary advice, continued glucose monitoring and treatment with insulin as indicated or (ii) usual care. We estimated the effect of fasting glucose on birth weight by the IV ratio estimate,^7^ but since change in fasting glucose was only assessed in the intervention arm we assumed that this was unchanged from baseline (pre-randomization) to later pregnancy in the control group. Considerable efforts were undertaken to ensure that neither the women themselves, nor their care-givers (nor research staff) of those randomized to routine care were aware that they had mild gestational diabetes, in order to ensure that they would not receive treatment for this. As part of normal routine care some women in the control group will have subsequently been identified as having gestational diabetes or impaired glucose tolerance, but there were marked differences between the two groups with respect to starting insulin therapy: two (0.4%) of the control group compared with 37 (7.6%) of the intervention group. However, maternal fasting glucose levels increase in the second and third trimesters of pregnancy. As a result the denominator of the IV ratio estimate (i.e. difference in fasting glucose by randomised group) is likely to have been an underestimate of the true difference and the IV estimate of the effect of fasting glucose on birth weight will be an exaggeration of any real causal effect.

Furthermore, in this approach there is potential for violation of the exclusion restriction criteria. The intervention consisted of dietary advice with addition of insulin if glucose monitoring suggested this was necessary. Insulin was only used in a minority of women (<8%), is not known to affect other (that glucose) characteristics and does not cross the placenta, so its use is unlikely to have resulted in violation of the exclusion restriction criteria. However, it is possible that the dietary advice (which was given to all women in the intervention arm), if followed, might reduce maternal blood pressure, adverse lipid profiles and other factors that could influence birthweight independently of glucose. The direction of any impact of this is unclear. In a recent MR study, we found evidence that lower systolic blood pressure is related to higher birth weight and no evidence for a clear effect of triglycerides or adiponectin on birth weight.^11^ Thus, if the dietary advice were followed and it resulted in lower maternal SBP this would potentially attenuate a true positive effect of fasting glucose on birth weight towards the null. In our MR study we were not able to test other lipids (beyond triglycerides) and nor were we able to test effects of fatty acids or amino acids. From observational evidenced, if the dietary changes reduced adverse lipids and fatty acids this might result in an exaggeration of a positive effect of glucose on birth weight.^13^

Considering both the bias due to assuming glucose did not change in the control group and possible violation of the exclusion restriction criteria (which could bias in either direction depending on whether blood pressure or lipids or other characteristics are most affected), we feel on balance this approach is likely to have biased results in a way that ***exaggerates*** the positive effect of glucose on birth weight. Our reason for suggesting this is that the bias from not having measured fasting glucose in the control group is very likely to be present, whereas the extent to which these women followed the dietary advice is unknown, as is the impact of following that advice on the exclusion restriction criteria. Though, as we note in the main paper, for this example we would conclude that additional studies are required to confidently answer this question.

The ***MR study*** that we used was from a publication of a study of the effect of maternal adiposity and associated traits, including fasting glucose, on birthweight that we were involved in.^11^ Genetic variants that have been shown to be robustly associated with fasting glucose in genome wide association studies (GWAS) of non-pregnant women and men were used and it was important that we were able to show that these were associated in a similar way to fasting glucose during pregnancy. This was done in a sub-sample of women with pregnancy fasting glucose samples and we found that the 13 fasting glucose increasing genetic variants used as instrumental variables had the same magnitude and direction of effect in pregnant women as those seen in the GWAS of non-pregnant women and men. We used a ratio IV estimate of the causal effect, in which the effect of each of the 13 fasting glucose variants on birthweight in our collaborative studies of 17,818 mother-offspring pairs was divided by their effects on fasting glucose from the published GWAS. We explored the strength of our IV, and the power that we had to detect minimal effect sizes that would be likely to be of public health importance and those results suggested that we were unlikely to have problems with weak instrument bias or lacked power.^11^ Genetic variants were selected that did not overlap with maternal adiposity or other adiposity-related trait variants in order to reduce the likelihood of bias due to violation of the exclusion restriction criteria because of genuine pleiotropy. We also explored heterogeneity between the IV ratio estimates for each variant and found that the point estimates were all very similar across the 13 variants in their IV ratio estimate (I^2^ = 0 and p = 0.43), suggesting violation of the exclusion restriction criteria is unlikely.

A clear path through which the exclusion restriction criteria might be violated is from mother’s genotype to offspring genotype and then an effect of offspring glucose genetic variants influencing their birth weight. To deal with this we adjusted for offspring genetic variants (the 13 fasting glucose variants that were used as instrumental variables) in the IV estimates. This did not notably alter the effect estimates. This adjustment could introduce some bias because once we adjust for offspring genotype we introduce a spurious association between father’s (which we do not have measured) and mother’s genotype. Father’s and mother’s genotype could also be related because of assortative mating. If father’s genotype (through their phenotype – ie. fasting glucose) is related to offspring birthweight then this could bias the IV estimate. We undertook sensitivity analyses to explore this which suggested that this might introduce a small amount of bias towards the null effect.

Overall, we think for the MR study the results are ***may be biased towards the null*** as a result of the adjustment made for offspring genotype (but as noted above without this adjustment there would have been a stronger bias away from the null).

**Illustrative example 3: What is the causal effect of having been breastfed on later body mass index?**

Over recent decades it has been suggested that being breastfed in infancy has life-long benefits across a range of health-related and disease outcomes, including that it protects against greater adiposity and obesity risk across life.^17,18^ Whilst the infant health benefits of being breastfed are not debated, determining whether these claims regarding longer-term benefit are valid is important.^17,18^ If there are causal beneficial effects long-term, for example on BMI, then this could point to mechanisms for preventing future ill-health. If, on the other hand, these claims are shown to be spurious (i.e. good quality evidence does not support them) then they damage the reputation of health research and might be used to argue against breast-feeding in general.

We combined our own knowledge of studies examining this question with literature searches and identified a large systematic review and meta-analysis of prospective cohort studies,^19^ a cross-cohort comparison,^20^ three within sibship studies (from two cohorts),^21-23^ and an RCT.^24,25^ We added a negative control study that we undertook for this paper, using data from the ALSPAC study.^26,27^ **Table 3** in the main paper summarises the approaches and the likely key sources of bias for the approaches used this example. Below we describe these in more detail.

For the ***multivariable regression*** analyses in prospective cohort studies we used results from a large published meta-analysis of summary data including up to 355,301 participants from over 35 studies (numbers varied in different analyses).^19^ A key source of bias in this approach is residual confounding since most of the studies that were included in the meta-analyses had adjusted for age and sex of the participant at the time of BMI assessment, but no other potential confounders. We considered maternal socioeconomic position (SEP) and BMI to be the most important confounders for this association and failure to account for these would result in an ***exaggerated*** estimate of the true effect. This is because mothers from more adverse socioeconomic positions in high income (Western) populations are less likely to breast feed and more likely to have fatter children, and women who are fatter are less likely to breast-feed and also have fatter children. That was explored within the meta-analysis which found that within the subgroup of studies able to adjust for more characteristics control for maternal SEP, BMI and smoking attenuated the inverse association to the null. We were able to explore this further in a triangulation framework using data from a published ***cross-context comparison*** that we had been involved in.^20,28^ We anticipated that in low and middle income countries (LMIC) breastfeeding would be more common and less socioeconomically patterned, whereas in high income countries, such as the UK there would be strong socioeconomic patterning with breast feeding being more common in women who were more educated and affluent. We demonstrated that this was true comparing equivalised SEP measurements with breastfeeding between participants from the ALSPAC study (UK), in which there were strong positive associations of SEP markers with breastfeeding and the Pelotas (Brazilian) study and also comparing ALSPAC to four additional LMICs (from India, South Africa, Guatemala and the Philippines). In the LMICs SEP markers were either unrelated to breastfeeding or related in the opposite direction (inverse) in comparison to the associations found in ALSPAC.^20,28^

In both ***within sibship*** studies breastfeeding was retrospectively reported by the mothers up to 16-years after the birth of their child. Misclassification of having been breastfed could have markedly ***attenuated*** any real causal effect towards the null.^21-23^ The expected attenuation towards the null of random misclassification would be greater in these within sibship analyses than between unrelated people because the sibship comparisons are on discordant pairs. In comparison to the concordant pairs, this discordant pairs will over-represent those in which one sibling is misclassified (i.e. the fact that you are looking at discordant pairs means you are picking up some for whom the mother has made an error in breastfeeding report for one – resulting in them being a discordant pair). Further ***attenuation*** could have resulted from measurement error in self-reported weight and height in the larger of the two within sibship studies.

The ***RCT*** analysed the intention to treat (by randomized groups) of a breast feeding promotion initiative that had been previously shown to increase breast feeding initiation and duration.^24,25^ No formal IV analysis was done to test the magnitude of effect of the difference in initiation and duration of breastfeeding, as it would have been impossible to separate the two and hence clearly interpret the findings, but this approach has the same principles as using IV analyses in and RCT. As with other such studies the main concern here would be violation of the exclusion restriction criteria (**Figure 1** in the main paper). It is possible that being randomised to the breastfeeding promotion arm of this trial had additional effects over and above the increase in breastfeeding that was observed, and that these might have biased results of breastfeeding effects on offspring BMI. For example, women randomised to the breastfeeding promotion arm might have adopted wider health promoting behaviours, including generally improving family diet and physical activity, which could then have been adopted by their children through childhood and adolescence, and those later postnatal effects (rather than breastfeeding) might drive any observed effect. This would spuriously ***exaggerate*** any true beneficial effect of breastfeeding on lower BMI. Having been breastfed might improve child intelligence, which might result in them making healthier choices and as a result having lower BMI. However, this would still be a ‘true’ effect of having been breastfed on BMI; the mechanisms of action would be (at least in part) via the impact of breast feeding on child’s intelligence. Thus, that would not bias the causal effect of having been breastfed on BMI.

Lastly, we undertook a ***negative outcome control study***, by examining the association of having been breastfed with home infestation by mice or pigeons, using data from the ALSPAC birth cohort collected when the children were aged 7-years (**Table s2**).^26,27^ Confounding by socioeconomic position (SEP) is a major concern in associations of breast-feeding with later maternal or offspring outcomes.^21^ Therefore we wanted a negative control outcome that is likely to be influenced by SEP but would not plausibly be influenced being breastfed. We also (ideally) wanted the outcome to be scaled similarly to the real outcome (BMI). We searched the databases of the ALSPAC and BiB cohorts (which we have access to) and were unable to find a continuously measured outcome that we felt would be a valid negative outcome. However, we did find two binary outcomes – parental report of house being invaded by mice and house being invaded by pigeons – that we *a priori* believed would be influenced by SEP, but would not be plausibly influenced by having been breast-fed. More affluent families and those from higher socioeconomic positions (SEP) are more likely to live in old (Victorian and Georgian) houses and these are more prone to have problems with mice, which is not something higher SEP families would be embarrassed to report. We therefore anticipated that there would be a positive (confounded) association of having been breast-fed and parental report of home invasion by mice (higher SEP being associated with increased likelihood of breast-feeding and home invasion by mice). By contrast we *a priori* assumed that report of home invasion by pigeons would be more common in lower SEP families (some of whom might keep pigeons as a hobby) and hence here we anticipated an inverse (confounded) association due to higher SEP being associated with increased likelihood of breast feeding but lower likelihood of pigeons in the house.

Because these were binary outcomes we compared associations with these negative control outcomes to childhood obesity (rather than BMI). We tested our assumptions regarding the relationship of SEP with each outcome and showed that markers of SEP and associated characteristics (household occupational social class, maternal education, maternal age, smoking and BMI in pregnancy) were generally associated in the directions anticipated (**Table s4**). Thus, a positive association of having been breast-fed with home invasion by mice and an inverse association with home invasion by pigeons (particularly if these were of a similar magnitude to that of the inverse association of having been breast-fed with obesity) would suggest that the ‘real’ inverse association of being breast-fed with obesity was confounded.

**Supplementary Table s1: Examples of using different epidemiological approaches to answer aetiological questions**

*Note; The number of examples provided for each approach and the detail in which they are described varies depending on how commonly used the approach is, how many examples are available in the literature and whether we felt several examples were required to illustrate key sources of bias for each approach in different contexts. See Table 1 in the main paper for a description of each approach, their assumptions and general potential key sources of bias*

| **Approach** | **Examples** |
| --- | --- |
| Randomised controlled trial | There are a large number of examples of well conducted RCTs that have established many important causes of disease. For example, well-conducted RCTs of statins have shown how they effectively reduce LDLc levels and CHD risk and well conducted RCTs of antihypertensives have shown how many of them effectively reduce blood pressure and CHD risk.^29-32^ RCTs have also shown important null results. For example, illustrating that antioxidant vitamins and HRT do not protect against CHD,^33-35^ and recently suggesting that elevating HDLc levels does not protect against CHD.^36^ There are also examples of interventions that have been implemented in clinical practice based on early RCTs, which with subsequent better conducted RCTs or systematic reviews of all RCTs, have been shown to have little or no benefit. For example, “prescriptions” of exercise are recommended as part of the treatment of depression based on early RCTs that included small numbers, inadequate concealment of random allocation and lack of blinding of outcome, whereas better conducted trials suggest at best weak benefit.^37,38^ |
| Multivariable analyses in observational data | *Smoking, LDLc and blood pressure*  Multivariable regression analyses in prospective cohort studies have provided evidence that cigarette smoking causes increased risk of lung cancer and premature mortality, that higher LDLc causes increased CHD and that higher blood pressure causes increased CHD and stroke.^5,39-45^ These observations have been supported by subsequent evidence, including secular downward trends in disease following reduced levels of smoking, cross-context comparisons, MR studies and, in the case of effects of LDLc and blood pressure on CHD, RCTs of effective interventions on these two exposures.^4,29-31,41,46-48^  *Hormone replacement, vitamins and HDLc*  In other examples findings from observational studies have been questioned when lack of support is found from other approaches. For example, the observational evidence that greater intakes of beta carotene, vitamin E, vitamin C or hormone replacement therapy (HRT) protected against CHD,^49-53^ resulted in widespread use of the vitamin supplements and prescriptions for HRT before RCTs showed no such protection.^32-35^ Time-varying effects, in the case of the relationship of HRT to CHD, and residual confounding for the vitamin and HRT associations, are plausible explanations for these discrepancies.^54-58^ By contrast, the failure of either RCTs or MR studies to support a causal effect of HDLc in protecting against CHD,^59-61^ is unlikely to be explained by residual confounding in the observational studies since LDLc and HDLc are likely to have similar confounders for their respective relationships to CHD. A plausible explanation is that HDLc is a strong predictor of CHD, but not a causal agent, because it is a proxy for the atherogenic properties of triglyceride.^62-64^ HDLc and triglycerides are inversely correlated and a consistent finding in prospective cohort studies has been that with mutual adjustment the inverse association of HDLc with CHD remains, whereas that of triglyceride levels with CHD attenuates to the null.^65^ This led to the suggestion that HDLc was causally related to CHD, but triglycerides were not. However, triglycerides are biologically very variable and hence a single measure is unlikely to reliably capture the atherogenic component that it potentially proxies for. By contrast HDLc has relatively little biological variability. From as far back as the mid-1980s investigators have shown these differences could explain why HDLc associations with CHD are robust to adjustment for triglycerides whereas triglyceride associations attenuate with adjustment for HDLc, and pointed out that this means one cannot distinguish which is the likely causal agent using conventional observational epidemiological approaches.^62-64^ |
| Cross-cohort comparison | *Long-term effects of having been breastfed*  A cross cohort comparison was undertaken comparing the association of being breastfed with BMI, blood pressure and IQ in a UK birth cohort (high income country (HIC)), a Brazilian (Low or Middle Income Country (LMIC)) birth cohort and, for BMI and blood pressure, pooled results from four LMIC cohorts.^20^ In LMICs it was reasoned that breast feeding would be more common and not as markedly patterned by SEP as it is in HICs. This was demonstrated for observed markers of SEP.^20^ In the LMICs being breastfed was not associated with offspring BMI or blood pressure, whereas it was in the UK cohort. By contrast, breastfeeding was positively associated with IQ in both the UK and Brazilian cohorts.^20^ These results suggest that being breastfed does not cause lower BMI or blood pressure and that its association with these in HIC was largely due to confounding, whereas being breastfed may have a beneficial causal effect on IQ. |
| Using different control groups | *Occupational and non-occupational causes of cancer*  The large Montreal case-control study of occupational and non-occupational causes of cancer has frequently used both general population and other cancer controls, with the former assumed to be more likely to be biased by selective information (recall bias) and the latter by selection bias due to some other cancers (unknowingly) also being influenced by the exposure under study. Data were collected from 3730 cancer patients (21 different cancers from 15 different sites) and 533 general population controls from the same population as the cancer cases. In one study the relationship of cigarette smoking with the 21 different cancer types was assessed using other cancer controls (this included all of the cancers except lung, bladder, oesophagus, pancreas, liver, kidney and stomach; no cancer represented more than 20% of the overall pool of cancer controls) and the general population controls.^66^ Results were similar for both types of control whether these showed an increased risk of the cancer with ever smoking (e.g. unadjusted odds ratios for population and cancer controls, respectively, were 16.1 and 16.4 for lung, 3.2 and 3.3. for oesophagus, 1.7 and 1.6 for stomach, 2.6 and 2.6 for bladder and 1.8 and 1.7 for pancreas cancers) or these showed no association (e.g. 1.1. and 1.1 for colon and 1.0 and 1.1 for rectal cancer). In a more recent use of the same study the association of occupational exposure to gasoline and diesel exhaust with lung cancer was examined.^67^ In that example there were similar null results for both types of control for exposure to gasoline exhaust only (any level): adjusted odds ratios: 0.8 (0.6, 1.0) and 0.9 (0.7, 1.1) for population and other cancer controls, respectively. However, the point estimates looked different for the two types of control with exposure to diesel exhausts (1.8 (0.9, 3.8) and 1.2 (0.8, 2.0), respectively for population and cancer controls). Whilst the population control might be biased due to recall it would be unlikely that that would selectively affect occupational diesel, but not gasoline, exhaust exposure, suggesting that the weaker association with cancer controls might be because diesel exhaust is (unknowingly) related to other cancers that were included in the pooled cancer controls. |
| Natural experiment | *John Snow, contaminated water and cholera*  A classical example of this approach is that of John Snow who demonstrated the relationship between household water supply and deaths from cholera in the mid-1800s.^68,69^ He described two cholera outbreaks in London one that occurred in 1849 and the second 1853 to 1854, between these two periods there was no evidence of a major cholera epidemic in London. Many of the districts in both outbreaks were supplied by one of two water companies (Lambeth Waterworks and Southwark and Vauxhall Water Company), with pipes from both companies going down the same streets in several neighbourhoods. In 1849 both companies obtained their water from the same area of the Thames, which was downstream of where raw sewage was emptied into the river. However, in 1852 the Lambeth company moved so that it obtained its water supply from much higher up the Thames where it was free from London sewage. Snow showed that during the 1853/54 epidemic death rates from cholera were much higher in households whose supply was from the Southwark and Vauxhall company compared with those from the Lambeth company, whereas no such difference had been apparent in the earlier epidemic.  *Cigarette smoking legislation and adult and child health*  The introduction of work-place and public smoking bans in some geographical areas has provided recent natural experiment opportunities for exploring the effect of such bans on smoking reduction and on health. Whilst some studies solely look within local areas, comparing population level outcomes before and after bans, others have looked at change in outcome rates between areas over time where one area has introduced a ban and another has not. A recent systematic review and meta-analysis of such legislation on adult health found evidence for reductions in population level cardiac, cerebrovascular and respiratory disease hospital admissions or deaths associated with the introduction of the legislation.^70^ The magnitude of the association was greatest for respiratory disease (compared with cardiac or cerebrovascular disease) and where the extent of the ban was more extensive; it did not change over time (suggesting an initial impact that neither decreased or increased long-term after the legislation). These findings suggest legislative banning of smoking in work and public places is effective and confirms other sources of evidence supporting a causal effect of cigarette smoking on these outcomes. For these studies in adults it is difficult, however, to ascertain how much of the reduction in outcomes is due to smokers quitting or reducing their consumption and how much is due to reduced passive smoking. Changes in rates of childhood outcomes in relation to cigarette smoking bans are more likely to reflect passive smoking and a systematic review and meta-analysis of perinatal and childhood outcomes found a reduction in hospital admissions for childhood asthma and diagnoses of preterm birth, but not for low birth weight in relation to smoking bans.^71^ |
| Within sibship analyses | *Maternal age and adverse perinatal outcomes*  Within sibship studies, in which the sibship groups are mothers, show that infants of mothers who gave birth before age 20 have similar risk of being small for gestational age or preterm as their first born maternal cousins (the infants of their mother’s sisters) who were born when their mothers were older.^72,73^ By contrast the same approach found that at older ages risk or these outcomes were increased with increasing age, and this was more so within maternal siblings than in conventional multivariable approaches between unrelated women.^73^ These findings suggest that confounding – most likely by background family socioeconomic position – explains at least some of the relationship of maternal age with perinatal outcomes such that at younger maternal ages it is exaggerated whereas at older maternal ages it is masked. In these studies analyses were restricted to first births so that individual level confounding by birth order could not explain the results. It is possible that by the age of 16 plus individual level confounders do influence the age at which different sisters chose to have their first birth; such confounding would exaggerate the association more so in these analyses than in analyses in unrelated individuals.  *Paternal age and offspring mental health*  Older paternal age can result in genetic mutations during spermatogenesis and it has been suggested that these can result in adverse mental health in offspring. In a large (2.6+ million) study of Swedish people older paternal age at birth was associated with marked increased risk of autism, attention-deficit/hyperactivity disorder, psychosis, bipolar disorder, suicide and substance abuse, as well as lower educational attainment within-sibships born to their father at different ages (i.e. sibling groups were offspring).^74^ These findings could be biased by individual level confounding by birth order, but similar magnitudes of association within-cousin groups, including when these were restricted to first born cousins argue against that.^74^  *Gestational diabetes and offspring adiposity*  Within-offspring sibship analyses suggest that exposure to maternal gestational diabetes is causally, via intrauterine mechanisms, related to offspring greater adiposity in childhood, adolescence and early adulthood.^13,75-77^ In these studies it is impossible to control for systematic individual level confounding by birth order, since for gestational diabetes most of the exposed siblings will have been the later born children. Birth size tends to increase with greater birth order, but the extent to which any association with later adiposity is mediated by birth size is unclear. Furthermore, studies that have examined the association of birth order with childhood or adult BMI have either found higher BMI in first born than later born or no association.^78^ |
| Use of IVs to test causal effects of intermediates in RCTs | Without describing it as an IV analysis Law and colleagues determined the causal effect of a 10mmHg reduction in systolic blood pressure and/or 5mmHg reduction in diastolic blood pressure on CHD using randomization to antihypertensives as the IV and the ratio (Wald) estimator.^6^ The compared the magnitude of effect between different classes of antihypertensive which act through different paths to each other and concluded that ‘pleiotropy’ was unlikely to have biased the findings as the results were the same for each of these different classes of antihypertensives. |
| Genetic Instrumental variable (Mendelian randomization) | Genetic variants that influence levels of LDLc demonstrate the causal effect of LDLc on CHD,^48,79,80^ consistent with findings of RCTs of LDLc lowering (statin) therapy.^29-31^ Indeed, MR suggests a stronger effect of life-long lower LDLc (based on the notion that the genetic variants would affect levels across the whole of life) than the effect of statins given in mid-life. By contrast, as discussed above, both recent MR studies, and RCTs, question any causal role of HDLc in protecting against CHD.^36,59,81^ MR has also been used to show that C-reactive protein is unlikely to causally affect CHD, insulin resistance and cardiometabolic risk factors,^82-86^ that greater adiposity is causally related to more adverse cardiometabolic risk factors,^87,88^ that greater alcohol consumption is likely to be detrimental to cardiovascular risk,^89-91^ and that adiponectin is not causally related to CHD,^92^ amongst an increasing number of examples using this approach. It is possible that some earlier MR studies that used a small number of genetic variants to test causal effects might have been biased by violation of the exclusion restriction criteria due to genuine (horizontal) pleiotropy. Comparisons of associations between several independent genetic instruments and use of new methods for testing and potentially controlling for genuine pleitropy^1,3^ are, and should be, increasingly used in MR studies.^92^ |
| Non-genetic Instrumental variable | *Offspring and causal effect of height and BMI on cause specific mortality*  In large Swedish record-linkage studies, son’s height and son’s BMI have been used as IVs to to test causal effects on all-cause and cause specific mortality.^93,94^ For many of the associations findings have been broadly consistent with those of conventional multivariable approaches. For example, the increased risk of cardiovascular disease with higher BMI and shorter height is observed in both conventional multivariable prospective cohort studies and also in these studies using son’s BMI/height as an IV for parents BMI/height, though in the IV analyses the positive association of BMI with cardiovascular mortality appeared stronger. However, it is possible that the causal estimates from both the IV and multivariable analyses are biased by residual confounding, for example family smoking patterns, and socioeconomic position (that may have been inadequately measured), as these are just as likely to be related to son’s height and BMI as they are to parents’ A major difference between the two approaches was in the relationship of BMI with respiratory diseases. Whilst large prospective cohort studies have found a strong inverse association of BMI with lung cancer and respiratory disease mortality,^95^ using son’s BMI as an IV there was no association of BMI with respiratory disease / lung cancer in men and a positive association in women. The stronger association with cardiovascular disease and the lack of support for an inverse association with respiratory disease and lung cancer when using son’s BMI as an IV is plausibly explained by reverse causality with these outcomes in conventional multivariable regression approaches.^94^ The authors argued in these studies that they were more concerned about using the IV to test the possibility of reverse causality than limit bias due to potential residual confounding.  *Economic shocks and civil conflict*  A study using variation in rainfall in Sub-Saharan Africa as an IV for the effect of economic shocks (sudden loss of income) on civil conflict concluded that acute economic shocks did cause civil conflict.^96^ The author considered whether variation in rainfall (the IV) could impact civil unrest and conflict via any paths other than through its effect on income (i.e. whether the exclusion restriction criteria might be violated). For example, he noted that very high rainfall could increase civil unrest through structural damage due to floods. Since there was a strong linear association of greater rainfall with higher income and the IV analyses showed a beneficial effect of higher income on reduced conflict this violation of the exclusion restriction criteria would tend to mean that the IV result was an underestimate of the true inverse association of income with civil conflict.^96^ |
| Exposure negative control study | *Paternal negative control exposures for intrauterine effects*  In studies that are concerned with the causal effect of maternal pregnancy exposures on offspring outcomes that are hypothesized to act via intrauterine mechanisms, father’s exposure (where available) is a potential valid negative exposure control, as confounders, and other sources of bias to causal estimates are likely to influence both parents similarly, but exposures in fathers would be unlikely to cause intrauterine effects. This approach has been used for at least six decades, being used by Jackson in the 1950s to explore whether the association of maternal diabetes on birth outcomes was an intrauterine causal effect or due shared genetic risk for diabetes and birth size, or biases (e.g. misclassification of diabetes, or confounding) ^13,97^ and by Yerushalmy in the 1960s and 70s to explore whether the association maternal smoking on birthweight and other outcomes was causal or due to bias that would be similar in both parents.^98,99^ In more recent examples negative control parental comparisons studies have found similar magnitudes of association between paternal smoking and offspring adiposity,^100^ cognitive function^101^ and bone mineral density,^102^ and of paternal BMI with later childhood offspring adiposity,^13,76^ to those seen with the same maternal exposures and offspring outcomes, suggesting confounding by shared familial SEP, lifestyles and genetic variation, rather than intrauterine causal effects explain these associations.^101^ In other examples such comparisons provide some evidence that maternal alcohol consumption in pregnancy results in reduced offspring academic attainment^103^ and that gestational diabetes causes greater adiposity in offspring.^13,104^ |
| Outcome negative control study | *HRT and CHD*  Prior to any RCTs of the effect of HRT on CHD, Diana Pettiti and colleagues cautioned against the suggestions that HRT prevented cardiovascular disease in women on the basis that the confounder-adjusted relative risk for cardiovascular disease, comparing ever to never use of HRT, was similar to that seen for accidental and violent deaths (negative outcome control).^105^ They could see no reason why the proposed biological mechanisms for protection against cardiovascular disease (improved lipid and coagulation profiles) would protect against accidents and violence, but they could see selection bias and/or confounding explaining the association of both.^105^  *Confounding by smoking in negative control studies*  Noting that many occupational cohorts do not have detailed (or any) data on smoking, Richardson and colleagues showed how a negative control study could be used to explore the extent to which confounding by smoking biased effects of occupational exposure to radon on lung cancer and of occupational exposure to radiation on lung cancer. They used two negative control outcomes – chronic obstructive pulmonary disease and cancer of the mouth, pharynx, larynx or oesophagus – assuming that confounding by smoking would be similar for both of these outcomes as it would for lung cancer but that there was no plausible causal effect of the exposures on these outcomes. For the effect of radon they were able to compare their findings to those of multivariable regression with control for smoking. Their findings suggested that radon was causally related to lung cancer and this association was not notably confounded by smoking; the latter conclusion was on the basis of observing a null association of radon with both negative controls and little change in the unadjusted association with lung cancer after adjustment for smoking. For radiation a positive association was found with both negative control studies that was weaker than the positive association seen with lung cancer. They used the negative control associations to estimate the magnitude of bias with lung cancer and concluded that confounding by smoking explained at least 18% of the observed effect of radiation on lung cancer, but acknowledged that they were unable to verify that.^106^ |

**Table s2: Results of studies used in triangulation to examine the effect of having been breast-fed on later BMI**

| **Approach** | **Measure of breast feeding** | **Measure of BMI** | **Analyses** | **Results** |
| --- | --- | --- | --- | --- |
| Conventional multivariable^19^ | Exclusive breast feeding | Kg/M^2^ | Mean BMI difference per category greater duration (age and sex adjusted). N = 32 studies | -0.08 (-0.10, -0.05) |
|  |  |  | Mean BMI difference per category greater duration (age and sex adjusted). N = 11 studies (subgroup of the 32 studies above that had data on move confounders) | -0.10 (-0.14, -0.06) |
|  |  |  | Mean BMI difference per category greater duration (age, sex, maternal SEP, BMI, smoking adjusted). N = 11 studies (same studies as above) | -0.01 (-0.05, 0.03) |
|  |  |  | Mean BMI difference exclusive breast-fed ≥ 8 months (age and sex adjusted). N = 3 studies (subgroup of the 32 studies) | -0.39 (-0.51, -0.26) |
|  |  |  | Mean BMI difference exclusive breast-fed ≥ 8 months (age, sex maternal SEP, BMI, smoking adjusted). N = 2 of the above 3 studies | -0.02 (95%CI or SE not provided) |
| Cross-cohort comparison^20,28^ | Not exclusive | Kg/M^2^ | Mean BMI difference per category greater duration (adjusted for family socioeconomic position indicators and child age) UK cohort. N = 4852 | -0.16 (-0.22, -0.09) |
|  |  |  | Mean BMI difference per category greater duration (adjusted for family socioeconomic position indicators and child age) LMIC cohorts. N = 10,912 | 0.04 (0.00, 0.08) |
| Within sibship (study 1)^21^ | Not exclusive | Age and gender standardized percentile | Mean BMI percentile difference per month of being breast-fed. Whole cohort between unrelated participants. N = 20,697 | -0.03 (-0.04, -0.02) |
|  |  |  | Mean BMI percentile difference per month of being breast-fed. Sibling cohort between unrelated participants analysis. N = 4,425 | -0.03 (-0.05, -0.01) |
|  |  |  | Mean BMI percentile difference per month of being breast-fed. Within sibship analyses. N = 1,046 (523 discordant sibship pairs) | 0.01 (-0.05, 0.07) |
|  |  |  | Mean BMI percentile difference ever vs never breast-fed. Whole cohort between unrelated participants. N = 20,697 | -0.41 (-0.55, -0.27) |
|  |  |  | Mean BMI percentile difference ever vs never breast-fed. Sibling cohort between unrelated participants. N = 4,425 | -0.34 (-0.85, 0.17) |
|  |  |  | Mean BMI percentile difference ever vs never breast-fed. Within sibship analyses. N = 1,046 (523 discordant sibship pairs) | 0.40 (-0.25, 1.05) |
| Within sibship (study 2)^23^ | Not exclusive | Age and gender standardized SD | Mean BMI percentile difference ever vs never breast-fed. Whole cohort between unrelated participants. N = 2,591 | -0.15 (-0.29, -0.01) |
|  |  |  | Mean BMI percentile difference ever vs never breast-fed. Sibling cohort between unrelated participants analysis. N = 976 | -0.15 (-0.37, 0.07) |
|  |  |  | Mean BMI percentile difference ever vs never breast-fed. Within sibship analyses. N = 118 (59 discordant sibship pairs) | -0.40 (-0.75, -0.05) |
| RCT^24,25^ | Intervention resulted in marked differences in ever, duration and exclusivity of breast feeding | Kg/M^2^ | Mean BMI difference intervention minus control arm at mean age 6.5 (N= 13,879) | 0.1 (-0.6, 1.0) |
|  |  |  | Mean BMI difference intervention minus control arm at mean age 15.5 (N= 13,866) | 0.16 (-0.02, 0.35) |
|  |  |  | Mean fat mass index difference intervention minus control arm at mean age 15.5 (N= 13,769) | 0.10 (-0.02, 0.22) |

**Supplementary Table s3: Negative outcome control study of the effect of being breastfed on childhood overweight/obesity. N = 6031**

| **Outcome** | **N (%) with outcome** | **Odds ratio (95% confidence interval) of outcome per unit or category of** | | | | | |
| --- | --- | --- | --- | --- | --- | --- | --- |
|  |  | **Maternal age (per year)** | **Manual vs non-manual social class** | **Mother not educated to university degree vs has university degree** | **Maternal BMI (per kg/m^2^)** | **Maternal smoking in pregnancy**  **(yes vs no)** | **Ever vs never breastfed^c^** |
| Home invasion by mice^a^ | 565 (9) | 1.08  (1.06, 1.10) | 0.69  (0.57,0.92) | 0.56  (0.46, 0.69) | 0.98  (0.96, 1.00) | 0.81  (0.63, 1.00) | 1.74  (1.34, 2.27) |
| Home invasion by pigeons^a^ | 382 (6) | 1.01  (0.99, 1.04) | 1.55  (1.19, 2.03) | 1.09  (0.82, 1.45) | 1.03  (1.00, 1.07) | 1.63  (1.29, 2.01) | 0.75  (0.58, 0.97) |
| **Obese^b^** | 235 (4) | 1.00  (0.97, 1.03) | 1.22  (0.85, 1.75) | 2.08  (1.33, 3.23) | 1.18  (1.15, 1.21) | 1.64  (1.21, 2.21) | 0.74  (0.54, 0.99) |

^a^ These outcomes were obtained from questionnaires completed by the child’s mother when the child was mean age 7 years

^b^ Based on measured weight and height when the child was mean age 7 years; we used obesity (rather than mean BMI as in the other studies) so that results could be compared to the binary negative control outcomes. Obesity was defined using international obesity task force criteria taking account of child’s exact age in 6-month categories.

^c^ Adjusted for confounders – maternal age, household social class, maternal education, maternal BMI, maternal smoking

Maternal report of their house being invaded by mice is more prevalent than child obesity and in general relates to confounders in the opposite direction to that seen for child obesity (higher social class, better educated women who do not smoke and have lower BMI are more likely to report mice in the home). Ever being breastfed is associated with this negative control outcome (home invaded by mice) but in the opposite direction to that seen for child obesity. However, this is the direction that one would expect from the differing way that confounders related to the two outcomes.

Maternal report of their house being invaded by pigeons has a similar prevalence to child obesity and the two have broadly similar associations with confounders. The adjusted association of ever versus never being breastfed with this negative control is the same as that seen for child obesity, suggesting that both are likely to be due to residual confounding.

The best explanation for all of these results is that they are all confounded – in particular by socioeconomic position and related health behaviours – and that adjustment for the observed confounders is insufficient to deal with this confounding.

**Figure s1: Sensitivity analyses of the two-sample Mendelian randomization effect of SBP on CHD**

Figure s1a

Figure s1b

Footnote to figure s1

Figure s1a shows the scatter plot of difference in mean systolic blood pressure (SBP) per SBP increasing allele against the difference in log odds of CHD per SBP increasing allele for 25 genetic variants related to SBP in the international collaboration of blood pressure (ICBP) genome-wide association collaboration.^8^ The results for the effect of these variants on CHD was taken from the CARDIoGRAM genome-wide association collaboration.^9^ Three different methods for pooling the data by fitting a regression line through the points that represent the IV effect estimated by each variant have been fitted: inverse variance weighting (IVW), MR-Egger^1^ and weighted median^3^. IVW has an intercept that is slightly higher than zero; the slope for this method is similar to that reported in Ference et al.,^4^ if its scale is changed to per 10mmHg lower SBP. The weighted median results are similar to those of the IVW method, but the MR-Egger results, which differs from the other two methods in weighting variant effects by the strength of the first stage regression taking account of how common the minor allele frequency is, suggests important levels of pleiotropy (a positive non-zero intercept) with little evidence of a causal effect of SBP on CHD when this is taken into account.

Figure s1b shows the funnel plot (the minor allele frequency first stage regression instrument strength plotted against the causal effect for each variant (SNP)). Both Figure 4a and 4b illustrate that the MR Egger results are influenced by one outlier (inside red dashed circle) which is associated with higher SBP but lower CHD. Further analyses confirm this is an outlier (**Figure s2**). When this outlier is removed the estimates for all three methods (IVW, weighted median and MR-Egger) are all consistent with each other and with a positive causal effect of SBP on CHD (**Figure** **s3**).

The analyses for these figures was undertaken by Jack Bowden (University of Bristol), with thanks. The authors take full responsibility for the interpretation of their results.

**Figure s2: Exploration of outlying genetic variant in the 2-sample MR of the effect of systolic blood pressure on coronary heart disease: Cooks distance and studentized residuals**

Figure S2a: MR-Egger

Figure s2b: Inverse variance weighting analyses

Footnote to Figure s2

Cooks distance and studentized residuals show one of the 25 SNPs is an outlier. The same SNP is identified in the MR-Egger and IVW analyses and is highlighted by a dashed red circle. The variant that is an outlier is rs17249754 in the ATPase plasma membrane Ca2+ transporting 1 (*ATP2B1*) gene, which is involved in intracellular calcium homeostasis, and is strongly associated with higher SBP but in the CARDIoGRAM consortium is associated with reduced CHD.

The analyses for these figures was undertaken by Jack Bowden (University of Bristol), with thanks. The authors take full responsibility for the interpretation of their results.

**Figure s3: Sensitivity analyses of 2-sample MR of effect of systolic blood pressure on coronary heart disease: Results with outlier SNP removed**

Figure s3a: Scatter plot

Figure s3b: Funnel plot

Footnote for Figure s3

Figure s3 shows the 2-sample Mendelian randomization analysis of SBP on CHD using 24 genetic variants. It differs from Figure s1 in that one of the 25 SNPs used in those analyses has been removed. The SNP that has been removed is rs17249754 in the *ATP2B1* gene, which Figures s2a and s2b illustrate is an outlier. With the exception of the removal of this outlier Figures s3a and 3b are showing identical methods and outputs to those shown in Figures s2a and s2b. MR-Egger Simex estimates have also been added to these figures; this account of regression dilution bias in the MR-Egger estimate. With the outlying variant removed the results from these three methods are now broadly consistent with each other.

The analyses for these figures was undertaken by Jack Bowden (University of Bristol), with thanks. The authors take full responsibility for the interpretation of their results.

**Supplementary references**

1. Bowden J, Davey Smith G, Burgess S. Mendelian randomization with invalid instruments: effect estimation and bias detection through Egger regression. *Int J Epidemiol* 2015; **44**(2): 512-25.

2. Lawlor DA. Two-sample Mendelian randomization: opportunities and challenges. . *International Journal of Epidemiology* 2016; **doi:10.1093/ije/dyw127**.

3. Bowden J, Davey Smith G, Haycock PC, Burgess S. Consistent Estimation in Mendelian Randomization with Some Invalid Instruments Using a Weighted Median Estimator. *Genet Epidemiol* 2016; **40**(4): 304-14.

4. Ference BA, Julius S, Mahajan N, Levy PD, Williams KA, Sr., Flack JM. Clinical effect of naturally random allocation to lower systolic blood pressure beginning before the development of hypertension. *Hypertension* 2014; **63**(6): 1182-8.

5. Lewington S, Clarke R, Qizilbash N, Peto R, Collins R. Age-specific relevance of usual blood pressure to vascular mortality: a meta-analysis of individual data for one million adults in 61 prospective studies. *Lancet* 2002; **360**(9349): 1903-13.

6. Law MR, Morris JK, Wald NJ. Use of blood pressure lowering drugs in the prevention of cardiovascular disease: meta-analysis of 147 randomised trials in the context of expectations from prospective epidemiological studies. *BMJ* 2009; **338**: b1665.

7. Greenland S. An introduction to instrumental variables for epidemiologists. *Int J Epidemiol* 2000; **29**(4): 722-9.

8. Ehret GB, Munroe PB, Rice KM, et al. Genetic variants in novel pathways influence blood pressure and cardiovascular disease risk. *Nature* 2011; **478**(7367): 103-9.

9. Consortium CAD, Deloukas P, Kanoni S, et al. Large-scale association analysis identifies new risk loci for coronary artery disease. *Nat Genet* 2013; **45**(1): 25-33.

10. Aschard H, Vilhjalmsson BJ, Joshi AD, Price AL, Kraft P. Adjusting for heritable covariates can bias effect estimates in genome-wide association studies. *Am J Hum Genet* 2015; **96**(2): 329-39.

11. Tyrrell J, Richmond RC, Palmer TM, et al. Genetic Evidence for Causal Relationships Between Maternal Obesity-Related Traits and Birth Weight. *JAMA* 2016; **315**(11): 1129-40.

12. Lawlor DA, Harbord RM, Sterne JAC, Timpson NJ, Davey Smith G. Mendelian randomization: using genes as instruments for making causal inferences in epidemiology. *Statistic in Medicine* 2008; **27**: 1133-63.

13. Lawlor DA. The Society for Social Medicine John Pemberton Lecture 2011. Developmental overnutrition--an old hypothesis with new importance? *Int J Epidemiol* 2013; **42**(1): 7-29.

14. Crowther CA, Hiller JE, Moss JR, McPhee AJ, Jeffries WS, Robinson JS. Effect of treatment of gestational diabetes mellitus on pregnancy outcomes. *N Engl J Med* 2005; **352**(24): 2477-86.

15. Wright J, Small N, Raynor P, et al. Cohort profile: The Born in Bradford multi-ethnic family cohort study. *Int J Epidemiol* 2013; **42**: 978-91.

16. Landon MB, Spong CY, Thom E, et al. A multicenter, randomized trial of treatment for mild gestational diabetes. *N Engl J Med* 2009; **361**(14): 1339-48.

17. Horta BL, Victora CG. Long-term effects of breastfeeging: A systematic Review. *WHO Library Cataloguing-in-Publication Data* 2013; **ISBN 978 92 4 150530 7**: Available at: <http://apps.who.int/iris/bitstream/10665/79198/1/9789241505307_eng.pdf>.

18. Goldberg G, Prentice A, Prentice A, Filteau S, Simondon K. Breast-feeding: early influences on later health. *Advances in Experimental Medicine* 2009; **639**: e-ISBN:978-1-4020-8749-3.

19. Owen CG, Martin RM, Whincup PH, Davey Smith G, Gillman MW, Cook DG. The effect of breast feeding on mean body mass index throughout the lifecourse; a quantitative review of published and unpublished observational evidence. *Amercian Journal of Clinical Nutrition* 2005; **82**: 1298-307.

20. Brion M-J, Lawlor DA, Matijasevich A, et al. What are the causal effects of breastfeeding on IQ, obesity and blood pressure? Evidence from comparing high-income with middle-income cohorts. *International Journal of Epidemiology* 2011; **40**: 670-80.

21. Evenhouse E, Reilly S. Improved estimates of the benefits of breastfeeding using sibling comparisons to reduce selection bias. *Health Serv Res* 2005; **40**(6 Pt 1): 1781-802.

22. Nelson MC, Gordon-Larsen P, Adair LS. Are adolescents who were breast-fed less likely to be overweight? Analyses of sibling pairs to reduce confounding. *Epidemiology* 2005; **16**(2): 247-53.

23. Metzger MW, McDade TW. Breastfeeding as obesity prevention in the United States: a sibling difference model. *Am J Hum Biol* 2010; **22**(3): 291-6.

24. Kramer MS, Matush L, Vanilovich I, et al. Long-term effects of prolonged and exclusive breastfeeding on child height, weight, adiposity, and blood pressure: New Evidence From A Large Randomized Trial. *American Journal of Clinical Nutrition* 2007; **86**: 1717-21.

25. Martin RM, Patel R, Kramer MS, et al. Effects of promoting longer-term and exclusive breastfeeding on adiposity and insulin-like growth factor-I at age 11.5 years: a randomized trial. *JAMA* 2013; **309**(10): 1005-13.

26. Boyd A, Golding J, Macleod J, et al. Cohort Profile: The 'Children of the 90s'--the index offspring of the Avon Longitudinal Study of Parents and Children. *Int J Epidemiol* 2013; **42**(1): 111-27.

27. Fraser A, Macdonald-Wallis C, Tilling K, et al. Cohort Profile: The Avon Longitudinal Study of Parents and Children: ALSPAC mothers cohort. *Int J Epidemiol* 2013; **42**(1): 97-110.

28. Fall CH, Borja JB, Osmond C, et al. Infant-feeding patterns and cardiovascular risk factors in young adulthood: data from five cohorts in low- and middle-income countries. *Int J Epidemiol* 2011; **40**(1): 47-62.

29. Collaboration BPLTT. Effects of different blood-pressure-lowering regimens on major cardiovascular events: results of prospectively-designed overviews of randomised trials. *Lancet* 2003; **362**(9395): 1527-35.

30. Shepherd J, Cobbe SM, Ford I, et al. Prevention of coronary heart disease with pravastatin in men with hypercholesterolemia. West of Scotland Coronary Prevention Study Group. *NEnglJMed* 1995; **333**(20): 1301-7.

31. Shepherd J, Blauw GJ, Murphy MB, et al. Pravastatin in elderly individuals at risk of vascular disease (PROSPER): a randomised controlled trial. *Lancet* 2002; **360**(9346): 1623-30.

32. Group HPSC. MRC/BHF Heart Protection Study of cholesterol lowering with simvastatin in 20536 high-risk individuals: a randomised placebo-controlled trial. *Lancet* 2002; **360**(9326): 7-22.

33. Manson JE, Hsia J, Johnson KC, et al. Estrogen plus progestin and the risk of coronary heart disease. *New England Journal of Medicine* 2003; **349**(6): 523-34.

34. Omenn GS, Goodman GE, Thornquist MD, et al. Effects of a combination of beta carotene and vitamin A on lung cancer and cardiovascular disease. *N Engl J Med* 1996; **334**(18): 1150-5.

35. Dietary supplementation with n-3 polyunsaturated fatty acids and vitamin E after myocardial infarction: results of the GISSI-Prevenzione trial. Gruppo Italiano per lo Studio della Sopravvivenza nell'Infarto miocardico. *Lancet* 1999; **354**(9177): 447-55.

36. Schwartz GG, Olsson AG, Abt M, et al. Effects of dalcetrapib in patients with a recent acute coronary syndrome. *N Engl J Med* 2012; **367**(22): 2089-99.

37. Mead GE, Morley W, Campbell P, Greig CA, McMurdo M, Lawlor DA. Exercise for depression. *Cochrane Database Syst Rev* 2009; (3): CD004366.

38. Chalder M, Wiles NJ, Campbell J, et al. Facilitated physical activity as a treatment for depressed adults: randomised controlled trial. *BMJ* 2012; **344**: e2758.

39. Doll R. Uncovering the effects of smoking: historical perspective. *StatMethods MedRes* 1998; **7**(2): 87-117.

40. Doll R, Peto R, Boreham J, Sutherland I. Mortality from cancer in relation to smoking: 50 years observations on British doctors. *Br J Cancer* 2005; **92**(3): 426-9.

41. Doll R, Peto R, Boreham J, Sutherland I. Mortality in relation to smoking: 50 years' observations on male British doctors. *BMJ* 2004; **328**(7455): 1519.

42. Peto R, Darby S, Deo H, Silcocks P, Whitley E, Doll R. Smoking, smoking cessation, and lung cancer in the UK since 1950: combination of national statistics with two case-control studies. *BMJ* 2000; **321**(7257): 323-9.

43. Kramer MS. Socioeconomic determinants of intrauterine growth retardation. *EurJ Clin Nutr* 1998; **52 Suppl 1**: S29-S32.

44. Manninen V, Tenkanen L, Koskinen P, et al. Joint effects of serum triglyceride and LDL cholesterol and HDL cholesterol concentrations on coronary heart disease risk in the Helsinki Heart Study. Implications for treatment. *Circulation* 1992; **85**(1): 37-45.

45. Perkovic V, Huxley R, Wu Y, Prabhakaran D, MacMahon S. The burden of blood pressure-related disease: a neglected priority for global health. *Hypertension* 2007; **50**(6): 991-7.

46. Chen ZM, Xu Z, Collins R, Li WX, Peto R. Early health effects of the emerging tobacco epidemic in China. A 16-year prospective study. *JAMA* 1997; **278**(18): 1500-4.

47. Jha P, Peto R. Global effects of smoking, of quitting, and of taxing tobacco. *N Engl J Med* 2014; **370**(1): 60-8.

48. Cohen JC, Boerwinkle E, Mosley TH, Jr., Hobbs HH. Sequence variations in PCSK9, low LDL, and protection against coronary heart disease. *N Engl J Med* 2006; **354**(12): 1264-72.

49. Osganian SK, Stampfer MJ, Rimm E, et al. Vitamin C and risk of coronary heart disease in women. *J Am Coll Cardiol* 2003; **42**(2): 246-52.

50. Rimm EB, Stampfer MJ, Ascherio A, Giovannucci E, Colditz GA, Willett WC. Vitamin E consumption and the risk of coronary heart disease in men. *N Engl J Med* 1993; **328**(20): 1450-6.

51. Stampfer MJ, Colditz GA. Estrogen replacement therapy and coronary heart disease: a quantitative assessment of the epidemiologic evidence. *PrevMed* 1991; **20**(1): 47-63.

52. Stampfer MJ, Colditz GA, Willett WC, et al. Postmenopausal estrogen therapy and cardiovascular disease. Ten-year follow-up from the nurses' health study [see comments]. *New England Journal of Medicine* 1991; **325**(11): 756-62.

53. Stampfer MJ, Hennekens CH, Manson JE, Colditz GA, Rosner B, Willett WC. Vitamin E consumption and the risk of coronary disease in women. *N Engl J Med* 1993; **328**(20): 1444-9.

54. Hernan MA, Alonso A, Logan R, et al. Observational studies analyzed like randomized experiments: an application to postmenopausal hormone therapy and coronary heart disease. *Epidemiology* 2008; **19**(6): 766-79.

55. Lawlor DA, Davey Smith G, Kundu D, Bruckdorfer KR, Ebrahim S. Those confounded vitamins: what can we learn from the differences between observational versus randomised trial evidence? *Lancet* 2004; **363**(9422): 1724-7.

56. Lawlor D, Davey Smith G, Bruckdorfer KR, Tilling K, Ebrahim S. Obervational versus randomized trial evidence. *Lancet* 2004; **364**: 754-5.

57. Lawlor DA, Ebrahim S, Kundu D, Bruckdorfer KR, Whincup PH, Davey Smith G. Vitamin C is not associated with coronary heart disease risk once life course socioeconomic position is taken into account: prospective findings from the British Women's Heart and Health Study. *Heart* 2005; **91**: 1086-7.

58. Lawlor DA, Davey SG, Ebrahim S. Socioeconomic position and hormone replacement therapy use: explaining the discrepancy in evidence from observational and randomized controlled trials. *Am J Public Health* 2004; **94**(12): 2149-54.

59. Voight BF, Peloso GM, Orho-Melander M, et al. Plasma HDL cholesterol and risk of myocardial infarction: a mendelian randomisation study. *Lancet* 2012; **380**(9841): 572-80.

60. Schwartz GG, Olsson AG, Barter PJ. Dalcetrapib in patients with an acute coronary syndrome. *N Engl J Med* 2013; **368**(9): 869-70.

61. Frikke-Schmidt R, Nordestgaard BG, Stene MC, et al. Association of loss-of-function mutations in the ABCA1 gene with high-density lipoprotein cholesterol levels and risk of ischemic heart disease. *JAMA* 2008; **299**(21): 2524-32.

62. Abbott RD, Carroll RJ. Interpreting multiple logistic regression coefficients in prospective observational studies. *Am J Epidemiol* 1984; **119**(5): 830-6.

63. Phillips AN, Smith GD. How independent are "independent" effects? Relative risk estimation when correlated exposures are measured imprecisely. *J Clin Epidemiol* 1991; **44**(11): 1223-31.

64. Durrington PN. Triglycerides are more important in atherosclerosis than epidemiology has suggested. *Atherosclerosis* 1998; **141 Suppl 1**: S57-62.

65. Di Angelantonio E, Sarwar N, Perry P, et al. Major lipids, apolipoproteins, and risk of vascular disease. *JAMA* 2009; **302**(18): 1993-2000.

66. Siemiatycki J, Krewski D, Franco E, Kaiserman M. Associations between cigarette smoking and each of 21 types of cancer: a multi-site case-control study. *Int J Epidemiol* 1995; **24**(3): 504-14.

67. Parent ME, Rousseau MC, Boffetta P, Cohen A, Siemiatycki J. Exposure to diesel and gasoline engine emissions and the risk of lung cancer. *Am J Epidemiol* 2007; **165**(1): 53-62.

68. Snow J. Cholera and the water supply in the South districts of London, in 1854. . *Journal of Public Health and Sanitary Review* 1856; **2**: 239-56.

69. Davey Simth G. Commentary: Behind the Broad Street pump: aetiology, epidemiology and prevention of cholera in mid-19th century Britain. *International Journal of Epidemiology* 2002; **31**: 920-32.

70. Tan CE, Glantz SA. Association between smoke-free legislation and hospitalizations for cardiac, cerebrovascular, and respiratory diseases: a meta-analysis. *Circulation* 2012; **126**(18): 2177-83.

71. Been JV, Nurmatov UB, Cox B, Nawrot TS, van Schayck CP, Sheikh A. Effect of smoke-free legislation on perinatal and child health: a systematic review and meta-analysis. *Lancet* 2014; **383**(9928): 1549-60.

72. Geronimus AT, Korenman S. Maternal youth or family background? On the health disadvantages of infants with teenage mothers. *AmJEpidemiol* 1993; **137**(2): 213-25.

73. Lawlor DA, Mortensen L, Andersen AM. Mechanisms underlying the associations of maternal age with adverse perinatal outcomes: a sibling study of 264 695 Danish women and their firstborn offspring. *Int J Epidemiol* 2011; **40**(5): 1205-14.

74. D'Onofrio BM, Rickert ME, Frans E, et al. Paternal age at childbearing and offspring psychiatric and academic morbidity. *JAMA psychiatry* 2014; **71**(4): 432-8.

75. Lawlor DA, Lichtenstein P, Langstrom N. Association of maternal diabetes mellitus in pregnancy with offspring adiposity into early adulthood: sibling study in a prospective cohort of 280,866 men from 248,293 families. *Circulation* 2011; **123**(3): 258-65.

76. Lawlor DA, Relton C, Sattar N, Nelson SM. Maternal adiposity--a determinant of perinatal and offspring outcomes? *Nat Rev Endocrinol* 2012; **8**(11): 679-88.

77. Dabelea D, Pettitt DJ. Intrauterine diabetic environment confers risks for type 2 diabetes mellitus and obesity in the offspring, in addition to genetic susceptibility. *J PediatrEndocrinolMetab* 2001; **14**(8): 1085-91.

78. Howe LD, Hallal PC, Matijasevich A, et al. The association of birth order with later body mass index and blood pressure: a comparison between prospective cohort studies from the United Kingdom and Brazil. *Int J Obes (Lond)* 2013.

79. Willer CJ, Sanna S, Jackson AU, et al. Newly identified loci that influence lipid concentrations and risk of coronary artery disease. *Nat Genet* 2008; **40**(2): 161-9.

80. Ference BA, Yoo W, Alesh I, et al. Effect of long-term exposure to lower low-density lipoprotein cholesterol beginning early in life on the risk of coronary heart disease: a Mendelian randomization analysis. *J Am Coll Cardiol* 2012; **60**(25): 2631-9.

81. Haase CL, Tybjaerg-Hansen A, Qayyum AA, Schou J, Nordestgaard BG, Frikke-Schmidt R. LCAT, HDL cholesterol and ischemic cardiovascular disease: a Mendelian randomization study of HDL cholesterol in 54,500 individuals. *J Clin Endocrinol Metab* 2012; **97**(2): E248-56.

82. Brunner EJ, Kivimaki M, Witte DR, et al. Inflammation, insulin resistance, and diabetes--Mendelian randomization using CRP haplotypes points upstream. *PLoSMed* 2008; **5**(8): e155.

83. Casas JP, Shah T, Cooper J, et al. Insights into the nature of the CRP-coronary event association using Medelian randomization. *International Journal of Epidemiology* 2006; **35**: 922-34.

84. Kivimaki M, Lawlor DA, Eklund C, et al. Mendelian randomization suggests no causal association between C-reactive protein and carotid intima-media thickness in the young Finns study. *Arterioscler Thromb Vasc Biol* 2007; **27**(4): 978-9.

85. Lawlor DA, Harbord RM, Timpson NJ, et al. The association of C-reactive protein and CRP genotype with coronary heart disease: findings from five studies with 4,610 cases amongst 18,637 participants. *PLoSONE* 2008; **3**(8): e3011.

86. Timpson NJ, Lawlor DA, Harbord RM, et al. C-reactive protein and its role in metabolic syndrome: mendelian randomisation study. *Lancet* 2005; **366**(9501): 1954-9.

87. Freathy RM, Timpson NJ, Lawlor DA, et al. Common variation in the FTO gene alters diabetes-related metabolic traits to the extent expected given its effect on BMI. *Diabetes* 2008; **57**(5): 1419-26.

88. Timpson NJ, Harbord R, G. DS, Zacho J, Tybjaerg-Hansen A, Nordestgaard BG. Does Greater Adiposity Increase Blood Pressure and Hypertension Risk?. Mendelian Randomization Using the FTO/MC4R Genotype. *Hypertension* 2009.

89. Lawlor DA, Nordestgaard BG, Benn M, Zuccolo L, Tybjaerg-Hansen A, Davey Smith G. Exploring causal associations between alcohol and coronary heart disease risk factors: findings from a Mendelian randomization study in the Copenhagen General Population Study. *Eur Heart J* 2013.

90. Chen L, Davey Smith G, Harbord RM, Lewis SJ. Alcohol Intake and Blood Pressure: A Systematic Review Implementing a Mendelian Randomization Approach. *PLoS Med* 2008; **5**(3): e52-doi:10.1371/journal.pmed.0050052.

91. Holmes MV, Dale CE, Zuccolo L, et al. Association between alcohol and cardiovascular disease: Mendelian randomisation analysis based on individual participant data. *BMJ* 2014; **349**: g4164.

92. Borges MC, Lawlor DA, de Oliveira C, White J, Horta BL, Barros AJD. The Role of Adiponectin in Coronary Heart Disease Risk: A Mendelian Randomization Study. *Circulation Research* 2016; **119**: 491-9.

93. Carslake D, Fraser A, Davey SG, et al. Associations of mortality with own height using son's height as an instrumental variable. *EconHum Biol* 2012; **doi:10.1016/j.ehb.2012.04.003**.

94. Davey Smith G, Sterne JA, Fraser A, Tynelius P, Lawlor DA, Rasmussen F. The association between BMI and mortality using offspring BMI as an indicator of own BMI: large intergenerational mortality study. *BMJ* 2009; **339**: b5043.

95. Whitlock G, Lewington S, Sherliker P, et al. Body-mass index and cause-specific mortality in 900 000 adults: collaborative analyses of 57 prospective studies. *Lancet* 2009; **373**(9669): 1083-96.

96. Miguel E. Economic Shocks and Civil Conflict: An Instrumental Variables Approach. *Journal of Political Economy* 2004; **112**: 725-53.

97. JACKSON WP. The prediabetic syndrome; large babies and the (pre) diabetic father. *J Clin Endocrinol Metab* 1954; **14**(2): 177-83.

98. Yerushalmy J. The relationship of parents' cigarette smoking to outcome of pregnancy--implications as to the problem of inferring causation from observed associations. *Am J Epidemiol* 1971; **93**(6): 443-56.

99. Yerushalmy J. Statistical considerations and evaluation of epidemiological evidence. In: James G, Rosenthal T, eds. Tobacco and Health. Springfield IL: Charles C. Thomas; 1962: 208-30.

100. Leary SD, Davey Smith G, Rogers IS, Reilly JJ, Wells JC, Ness AR. Smoking during Pregnancy and Offspring Fat and Lean Mass in Childhood. *Obesity* 2006; **14**(12): 2284-93.

101. Lawlor DA, Leary S, Davey Smith G. Theoretical underpinning for the use of intergenerational studies. In: Lawlor DA, Mishra GD, eds. Family matters Designing, analysing and understanding family-based studies in life course epidemiology. Oxford: OUP; 2009: 13-38.

102. Macdonald-wallis C, Tobias JH, Davey Simth G, Lawlor DA. Parental smoking during pregnancy and offspring bone mass at age 10 years: findings from a prospective birth cohort. *Osteoporosis International* 2011; **22**: 1809-19.

103. Alati R, Davey Smith G, Lewis SJ, et al. Effect of prenatal alcohol exposure on childhood academic outcomes: contrasting maternal and paternal associations in the ALSPAC study. *PLoS One* 2013; **8**(10): e74844.

104. Dabelea D, Hanson RL, Lindsay RS, et al. Intrauterine exposure to diabetes conveys risks for type 2 diabetes and obesity: a study of discordant sibships. *Diabetes* 2000; **49**(12): 2208-11.

105. Petitti DB, Perlman JA, Sidney S. Postmenopausal estrogen use and heart disease. *N Engl J Med* 1986; **315**(2): 131-2.

106. Richardson DB, Laurier D, Schubauer-Berigan MK, Tchetgen Tchetgen E, Cole SR. Assessment and indirect adjustment for confounding by smoking in cohort studies using relative hazards models. *Am J Epidemiol* 2014; **180**(9): 933-40.
